# Quantifying Public Engagement With Science and Malinformation on COVID-19 Vaccines: Cross-Sectional Study

**DOI:** 10.2196/64679

**Published:** 2025-03-21

**Authors:** David Robert Grimes, David H Gorski

**Affiliations:** 1 TCD Biostatistics Unit School of Medicine Trinity College Dublin Dublin Ireland; 2 School of Physical Sciences Dublin City University Dublin Ireland; 3 Michael and Marian Ilitch Department of Surgery Wayne State University Detroit, MI United States; 4 Barbara Ann Karmanos Cancer Institute Detroit, MI United States

**Keywords:** misinformation, altmetrics, disinformation, malinformation, public engagement, medical journals, medicoscientific, public health, altmetric analysis, comparative analysis, social media, Twitter, vaccine, digital health, mHealth, mobile health, health informatics

## Abstract

**Background:**

Medical journals are critical vanguards of research, and previous years have seen increasing public interest in and engagement with medicoscientific findings. How findings propagate and are understood and what harms erroneous claims might cause to public health remain unclear, especially on publicly contentious topics like COVID-19 vaccines. Gauging the engagement of the public with medical science and quantifying propagation patterns of medicoscientific papers are thus important undertakings. In contrast to misinformation and disinformation, which pivot on falsehood, the more nuanced issue of malinformation, where ostensibly true information is presented out of context or selectively curated to cause harm and misconception, has been less researched. As findings and facts can be selectively marshaled to present a misleading picture, it is crucial to consider this issue and its potential ramifications.

**Objective:**

This study aims to quantify patterns of public engagement with medical research and the vectors of propagation taken by a high-profile incidence of medical malinformation.

**Methods:**

In this work, we undertook an analysis of all altmetric engagements over a decade for 5 leading general-purpose medical journals, constituting approximately 9.8 million engagements with 84,529 papers. We identify and examine the proliferation of sentiment concerning a high-profile publication containing vaccine-negative malinformation. Engagement with this paper, with the highest altmetric score of any paper in an academic journal ever released, was tracked across media outlets worldwide and in social media users on Twitter (subsequently rebranded as X). Vectoring media sources were analyzed, and manual sentiment analysis on high-engagement Twitter shares of the paper was undertaken, contrasted with users’ prior vaccine sentiment.

**Results:**

Results of this analysis suggested that this COVID-19 scientific malinformation was much more likely to be engaged and amplified with negative by vaccine-negative Twitter accounts than neutral ones (odds ratio 58.2, 95% CI 9.7-658.0; *P*<.001), often alluding to the ostensible prestige of medical journals. Malinformation was frequently invoked by conspiracy theory websites and non-news sources (71/181 citations, 39.2%) on the internet to cast doubt on the efficacy of vaccination, many of whom tended to cite the paper repeatedly (51/181, 28.2%).

**Conclusions:**

Our findings suggest growing public interest in medical science and present evidence that medical and scientific journals need to be aware of not only the potential overt misinformation but also the more insidious impact of malinformation. Also, we discuss how journals and scientific communicators can reduce the influence of malinformation on public understanding.

## Introduction

The growing dominance of social media as a news source has resulted in not only widespread engagement with science but also a perpetuation of medical misinformation in fields ranging from dementia [1] to cardiology [2] and cancer [3,4] and beyond. Vaccines have been a topic of misinformation for centuries [5], with antivaccine propaganda being a leading contributor to declining vaccine uptake worldwide [6], and in 2019, the World Health Organization named vaccine hesitancy a top threat to public health, with COVID-19 vaccines being a recent major focus for an abundance of misinformation [7,8].

Misinformation undermines trust in medical science [9], leading to calls for medical journals to be more proactive in combatting health fiction, pseudoscience, and misconceptions. Armstrong and Naylor [10] argue that medical journals have a critical role in addressing medical misinformation by containing the dissemination of misinformation, identifying falsehoods and their purveyors, and striving to avoid false balance and legitimization of false information. They suggest that medical journals have a unique role in debunking myths and facilitating health-specific inoculation and education. Medical journals, as repositories of trustworthy information, have a central role to play, as “...the ability to credit information from scientific journals, and...to discredit information without such sources, is perhaps the most conventional countermeasure to misinformation” [11]. Directing the public to reliable sources of information, including medical journals, has been identified as an urgent undertaking in combatting COVID-19 misinformation [12].

Medicoscientific journals are not only trusted gatekeepers for reliable findings but are also far from infallible. Moreover, peer-reviewed medical science is no longer read and propagated solely by medical scientists but is widely disseminated by social media influencers and the wider public. The pandemic era has witnessed several high-profile retractions resulting from flawed analysis or even outright fabricated data. As Toth et al [13] note, “Medical journals not properly ‘vetting’ and eventually publishing high-impact papers that turn out to be based on unverified data can misguide providers as well as the public. Information on the disease that was previously restricted to medical professionals is now available to the broad public by the swipe of a screen, just like any other newsreel.” Accordingly, findings can be used in misinformation, disinformation, and malinformation, as defined in Table 1.

**Table 1:** Council of Europe definitions [14].

Term	Definition	Characteristics
Misinformation	“Information that is false, but not created with the intention of causing harm”	False information spread inadvertently or intentionally.
Disinformation	“Information that is false and deliberately created to harm a person, social group, organization or country”	False information introduced and spread with the intent to mislead or confound.
Malinformation	“Information that is based on reality, used to inflict harm on a person, organization or country”	Nominally true information is misleadingly presented or curated with the intention to deceive or mislead.

Unlike misinformation and disinformation, malinformation does not depend on false information, but instead leverages plausible and accurate—or at least arguably accurate—information presented either without context or in an incorrect context to lead those encountering it to false or misleading impressions and conclusions. When encountered without cognizance of crucial background or context, malinformation can create perceptions at odds with reality and cause harm to individuals, organizations, and collective understanding. In medical journals, malinformation can emerge through several mechanisms.

- Authors curate information out of context, with the net result of presenting a misleading impression of findings that are undetected by peer reviewers and editorial staff.
- Distortion of published research findings by third-party vested interests to cherry-pick or misrepresent legitimate published medical science to create a narrative at odds with the scientific evidence base.
- The synergy between these mechanisms.

To date, there has been comparatively little research done on quantifying engagement with medical science or the problems of malinformation in a medical context. Altmetrics measure the broader impact of scholarly works by analyzing social media and media engagement beyond traditional citations. They track mentions on social media, news, blogs, policy documents, and academic tools like Mendeley. Data are gathered using persistent identifiers (typically DOIs), which are weighted by source to generate composite altmetric scores. Altmetrics provide immediate insights into public and academic attention, complementing traditional metrics, a barometer of how research is understood and engaged with by the public on social and conventional media [15]. As they yield data complementary to citation-based metrics, tracking social media engagement, press coverage, and social media discussion, we took a novel approach of analyzing altmetrics as an indicator of engagement with medical science and the spread of potential malinformation, providing insight into how claims in medical journals are consumed in context and potential public health impacts.

## Methods

### Quantifying Engagement Metrics

**Figure 1:**
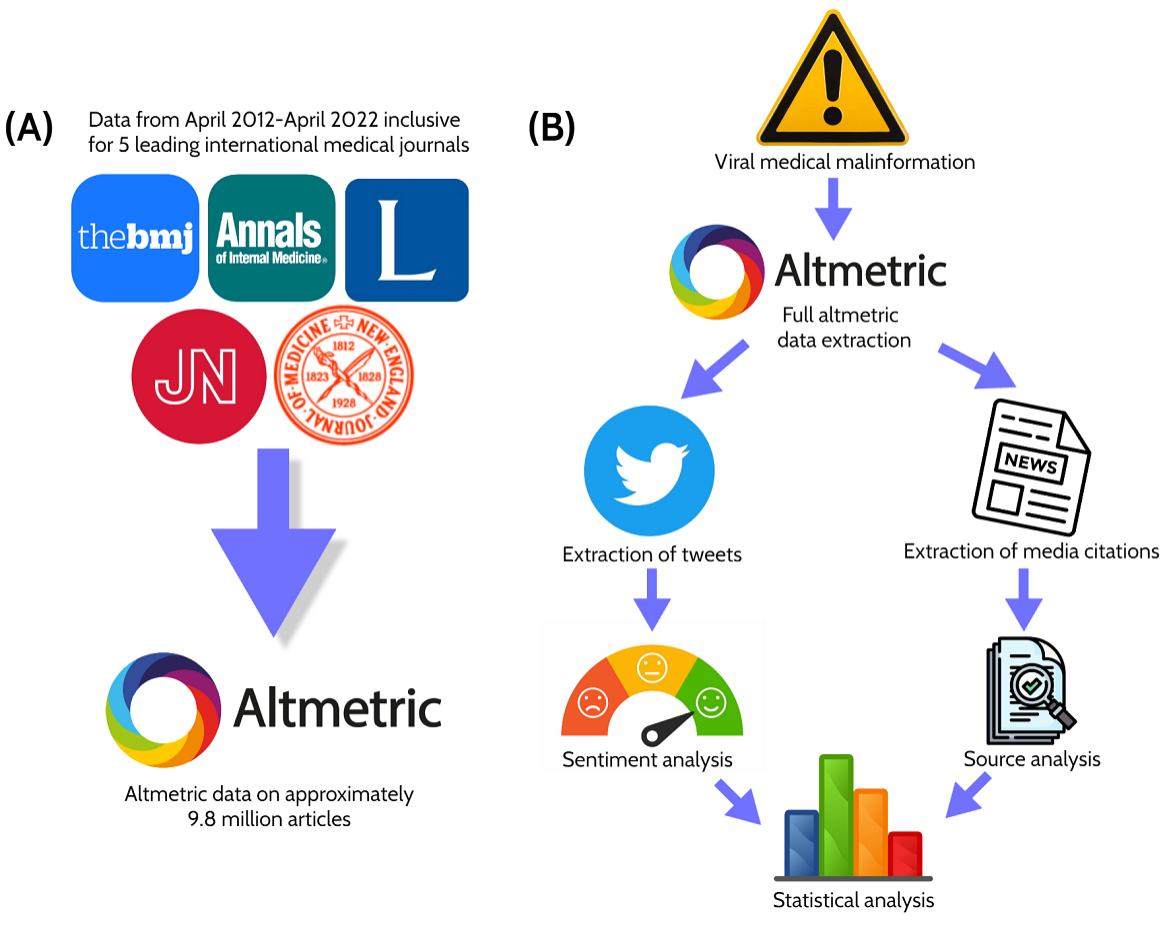
(A) Extraction of altmetric data for papers published over 10 years for 5 major journals, yielding approximately 9.8 million engagements with 84,529 papers. (B) Deep analysis of identified malinformation, including tweets and media citations.

Raw historical altmetric records for 5 leading medical journals (*New England Journal of Medicine*, *The Lancet*, *JAMA*, *Annals of Internal Medicine*, and *British Medical Journal*) for all papers published between April 2012 and April 2022 were exported from Altmetric Explorer. The CSV files for each journal included altmetric attention scores and publication dates for all papers in the 10-year interval, which were extracted and imported into MATLAB (MathWorks) on May 10, 2022. These journals were selected as a representative sample of respected broad-interest medical journals as opposed to more specialist offerings [16-18] to capture the typical dynamics of public engagement with general medical science over a long interval. Data were subsequently pooled and sorted to produce a hierarchical ranking of all altmetric scores. From this, the Lorenz curve and Gini coefficient were derived to ascertain the relative importance ranking of papers in terms of public engagement. Mathematical outlines are provided in Multimedia Appendix 1, and the process is illustrated in Figure 1A.

### Identification and Quantification of Potential Misinformation or Malinformation

A specific breakdown of the detailed altmetric mentions in a full analysis of a single medical journal publication from November 2021 [19] was also undertaken. For this paper, which garnered the highest altmetric score ever achieved in any scientific journal in any field published to the date of extraction, specific altmetric data were extracted, including all media mentions, outlet names, and linking tweets. At the time of data extraction, direct Twitter (subsequently rebranded as X) engagement with the paper yielded 165,720 tweets from 81,861 users with an upper bound of 11,094,945 followers, 181 media mentions, and a total altmetric score of 45,844. This investigative piece suggested that Pfizer vaccine trials were compromised by the unblinding of subjects and data falsification. Serious issues with this reporting were detected almost immediately after publication by independent fact-checks [20], medical press [21], and medical writers [22]. Criticisms focused chiefly on the paucity of evidence presented to support the statements in the paper, which were largely speculative and extrapolated unverified reported problems at one contract research organization center in Texas to all 153 centers worldwide.

No evidence was presented for the incendiary charge of data falsification, and ostensible problems at a single site were extrapolated to the entire clinical trial 24 times in the paper. Several other problems in the reporting and framing were identified, outlined in Multimedia Appendix 1. As the paper’s assertions, based on unfounded extrapolation, were likely harmful to public perception of Pfizer and of vaccine science more broadly, the European Council’s definition of malinformation (“information that is based in reality, but is used to inflict harm on a person, organization or country” [14]) appears most fitting here, given that real potential issues with a single contract research organization being contracted by Pfizer were used as a basis for unevidenced claims that Pfizer “falsified data” and “unblinded patients,” despite such assertions being at best speculative extrapolation. This could arguably be deemed misinformation too, a point expanded upon in the *Limitations* section. As this paper has by far the highest altmetric score of any paper in a biomedical journal (or any other scientific work in any field) ever published, extended altmetric analysis was performed to analyze its propagation and reception, including a breakdown of media sources, described in the later section and illustrated in Figure 1B.

### Propagation Analysis (Media Outlets)

For the extended analysis, all media mentions were systematically exported from Altmetric Explorer. Mentions with the header “News Story” were extracted into MATLAB. Sources were categorized as news stories (recognized media outlets and investigative media organizations) and aggregators, collectively deemed news sources. Mentions from non-news conspiracy theories or propaganda sites were also counted, as were sources from Russian state media outlets and blogs, collectively deemed non-news sources (objective criteria for demarcation are provided in Multimedia Appendix 1). A nonparametric right-tailed Wilcoxon rank sum test was performed to investigate the hypothesis that non-news sources cited the paper with greater frequency than reliable outlets.

### Sentiment Analysis (Social Media)

To ascertain how this paper was understood in a wider context, a Twitter search strategy was implemented for manual sentiment analysis for tweets relating to the paper in question. These were taken from the altmetric data and from an additional search containing the primary terms “Pfizer” and “BMJ,” with any of the secondary terms (“whistleblower,” “whistle,” “fraud,” “trials,” and “Ventavia”). All tweets with greater than 50 engagements were selected for manual sentiment analysis. For any account hosting such tweets, an advanced search was conducted on their username for all tweets with the terms “vaccine,” “vaccines,” ”COVID,” ”Pfizer,” ”hoax,” or “lies.” The 100 most recent tweets for each user under these criteria were exported. Users whose mention of vaccines in this set was solely negative or propagated discredited antivaccine propaganda were deemed as having vaccine-negative attitudes, while users whose set included mixed or positive vaccine stances were deemed non-negative. When the sentiments were ambiguous, cases were recorded as non-negative. Follower counts, engagement, and reach were also computed, with full data given in the Multimedia Appendix 1. A 2×3 Fisher exact test was used to test the hypothesis that vaccine-negative accounts were far more likely to embrace the paper as evidence against COVID-19 vaccination than non-negative accounts. All raw data are available in Multimedia Appendix 1.

### Ethical Considerations

This study involved analysis of publicly accessible tweets, which are considered public behavior data not requiring ethical or institutional board review. No informed consent was sought, as data analyzed are publicly available and no interaction with the authors of the tweets occurred. The data were handled in compliance with ethical guidelines for research using publicly available data, with anonymized tweets—to ensure that individuals cannot be reidentified—available in Multimedia Appendix 1 and unredacted versions available to reviewers and editors. No identifiable images or data from individual users are included in this paper or Multimedia Appendix 1. Where tweet content is cited verbatim, usernames and identifying details have been removed or generalized to protect privacy. An exception was made for a tweet authored by Robert F Kennedy Jr, a public figure, as it represents a public statement made in his capacity as a public advocate.

## Results

### Quantifying Engagement Metrics

**Figure 2:**
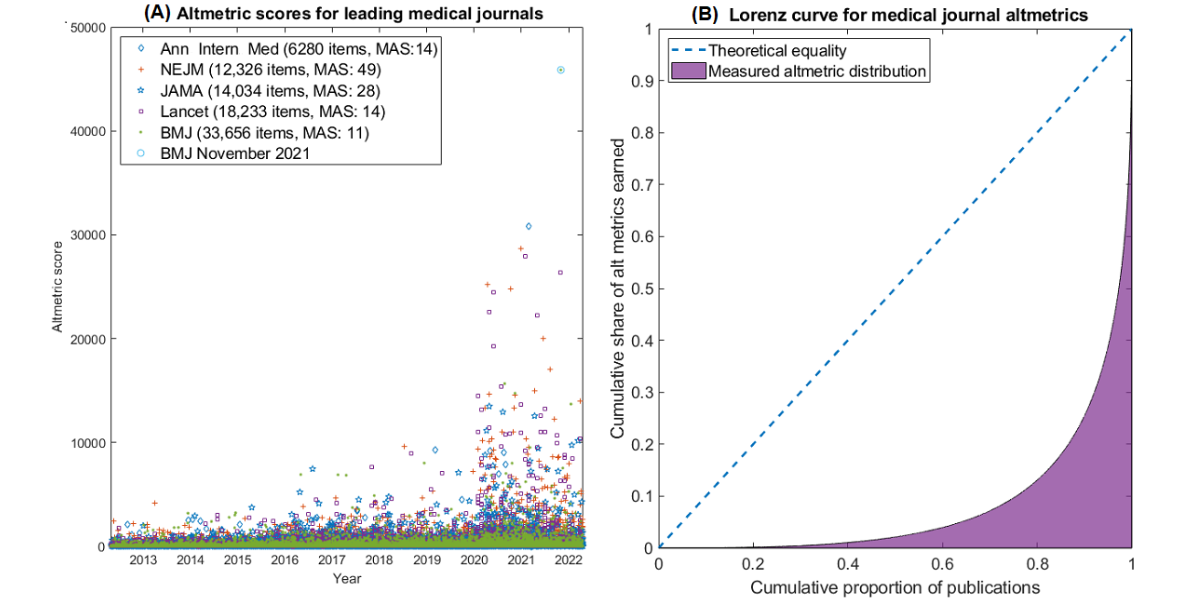
(A) Altmetric scores for every paper published in 5 leading medical journals between April 26, 2012, and April 26, 2022. The MAS and number of publications are given in the legend. (B) Lorenz curve for medical altmetrics (Gini coefficient=0.835). Ann Intern Med: Annals of Internal Medicine; BMJ: British Medical Journal; MAS: median altmetric score; NEJM: New England Journal of Medicine.

Figure 1A depicts altmetric trends for 5 major medical journals in the decade from April 26, 2012, to April 26, 2022, for 85,529 papers, depicting growing public interest with time. It is worth noting, however, that interest is greatly skewed. Figure 1B depicts the Lorenz curve and derived Gini coefficient of 0.835, demonstrating that altmetrics are driven by a small proportion of papers, quantified in Table 2. For all 5 included journals, the median altmetric scores of papers were also calculated (Figure 2A). As the safety and efficacy of COVID-19 vaccines were a worldwide topic of interest both on social media and traditional media, Table 3 gives the altmetric scores from the most highly discussed research works on vaccine efficacy trials for Moderna, Pfizer, and AstraZeneca vaccines.

**Table 2:** Altmetric statistics (medical papers).

Percentile of papers (%)	Proportion of total altmetrics (%)
Top 1	34.2
Top 0.5	25.6
Top 0.1	11.1
Top 0.01	2.37

**Table 3:** Altmetric scores for COVID-19 vaccine studies.

Date	Vaccine	Title	Journal	Altmetric score
July 2020	Moderna	An mRNA Vaccine against SARS-CoV-2—preliminary report	*NEJM* ^a^	14,325
August 2020	AstraZeneca	Safety and immunogenicity of the ChAdOx1 nCoV-19 vaccine against SARS-CoV-2: a preliminary report of a phase 1/2, single-blind, randomized controlled trial	*Lancet*	15,254
December 2020	Pfizer	Safety and efficacy of the BNT162b2 mRNA Covid-19 Vaccine	*NEJM*	29,151
November 2021	Pfizer	Covid-19: Researcher blows the whistle on data integrity issues in Pfizer’s vaccine trial	*BMJ* ^b^	45,844

^a^NEJM: New England Journal of Medicine.

^b^BMJ: British Medical Journal.

### Propagation Analysis (Media Outlets)

**Figure 3:**
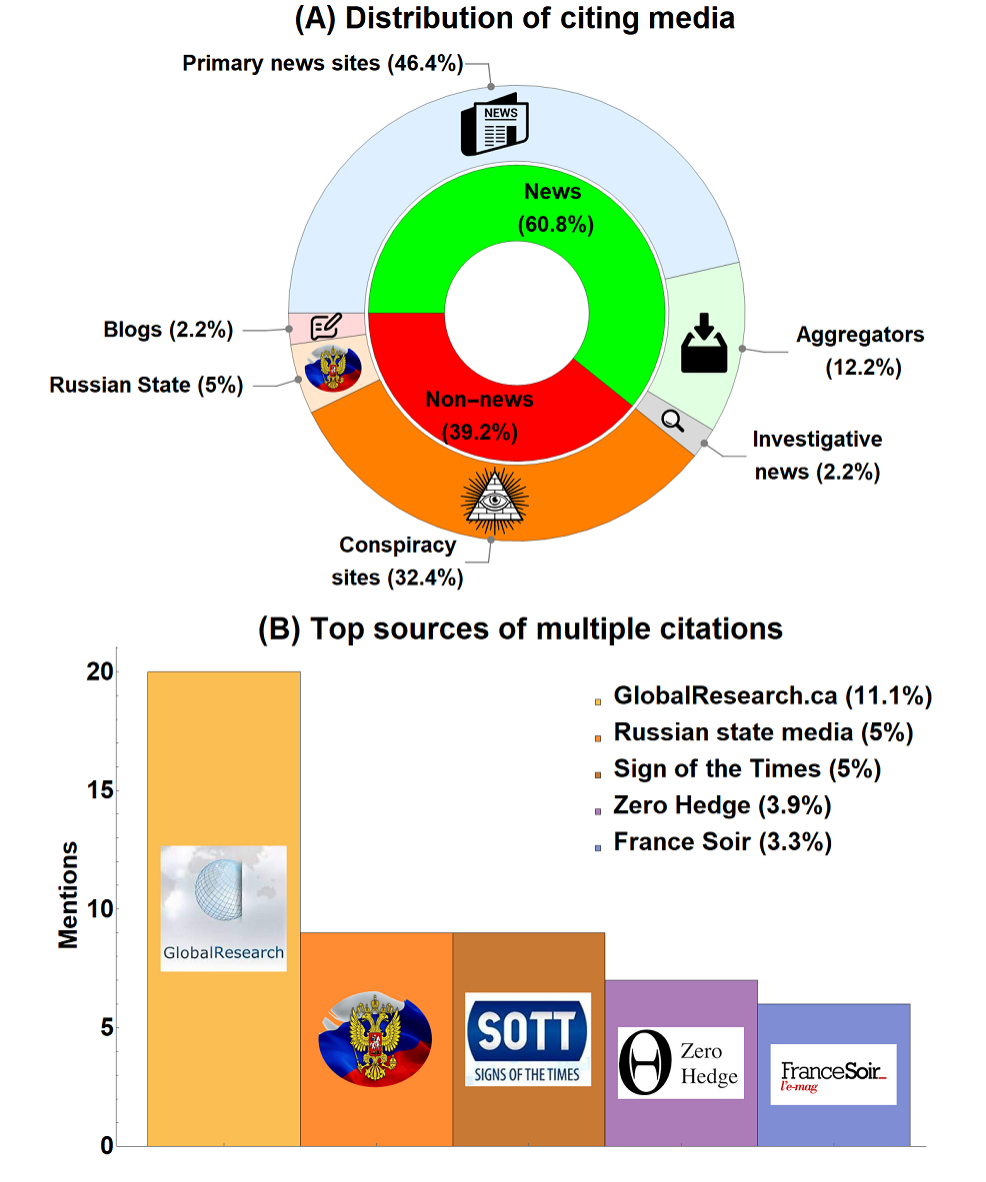
(A) Quantification of news-related altmetrics for case study from publication to April 26, 2022, inclusive. (B) Sources with the highest frequency of paper mentions. Legend indicates what proportion of total media citations for which these sources account.

Figure 3A depicts the media distribution of the paper from publication to April 26, 2022, where 71 (39.2%) out of all 181 media citations of the paper came from non-news sites. Figure 3B shows the cumulative mentions from the 5 sources that cited the paper multiple times, all of which were non-news media and conspiracy site sources, with explicit classification means of reporting sites given in Multimedia Appendix 1. These 5 outlets accounted for 51 (28.2%) of the 181 total media mentions alone. A nonparametric right-tailed Wilcoxon rank sum test was performed to investigate the hypothesis that illegitimate non-news sources cited the paper with greater frequency than reliable outlets; for both cases where all Russian outlets were individually counted or pooled as a single entity, the result was highly significant (*P*<.001). This strongly suggests the paper was disproportionally embraced by partisan outlets with documented histories of antivaccine sentiment rather than conventional media.

### Sentiment Analysis (Social Media)

Our Twitter search strategy yielded 75 tweets from 68 unique users, of which vaccine-negative accounts (52/68, 76%) yielded an upper-bound direct audience of 3,701,281, whereas non-negative accounts (16/68, 24%) had an upper-bound direct audience of 979,198, a 3.78-fold reach difference. Vaccine-negative accounts included the Russian state Sputnik vaccine account (more than 1 million followers, denoted as a vaccine-negative account despite its ostensible purpose due to its disparagement of other vaccines and spreading conspiracy theories about them) and Robert F Kennedy Jr’s account (420,900 followers at the time of analysis, widely criticized for vectoring virulent antivaccine propaganda). The non-negative account with the greatest number of followers was the journal’s official Twitter account (489,800 followers).

Table 4 depicts the sentiment of high-engagement tweets, distinguishing between shares of the story without caveats or with negative editorialization (including terms like “fraud,” “criminal,” “corruption,” “enemy,” “fake,” and “scandal”) versus those which reported without negative editorialization with caveats or actively rebutted the paper. A Freeman-Halton extension of the Fisher exact test for 2×3 case yields *P*<.001, strongly supporting the hypothesis that vaccine-negative accounts were far more likely to embrace the paper as evidence against COVID-19 vaccination than non-negative accounts. Vaccine-negative accounts were far more likely to share the paper with negative editorialization (odds ratio 58.2, 95% CI 9.7-658.0).

**Table 4:** Sentiment division on paper.

Preexisting vaccine sentiment	Neutral or caveated presentation	Negative editorialization	Refutation
Non-negative	8	2	6
Negative	5	47	0

## Discussion

### Principal Findings

The results of our analysis suggest a growing public interest in medical science and highlight a clear instance of how even a dubious finding in medical journals can readily be propagated, buoyed by the veneer of legitimacy that comes from being published in a respected journal. In some respects, this finding is not unexpected. The now infamous case of Andrew Wakefield’s fraudulent *Lancet* paper in 1998 led to an enduring confidence crisis over the measles-mumps-rubella vaccine [23], and more recently, dubious claims in medical journals about the efficacy of ivermectin [24] and hydroxychloroquine [25] in treating COVID-19 wrongly led millions to ineffective treatments for the condition; in the case of ivermectin, it even led people to purchase the drug from veterinary suppliers. In these cases, however, the primary information itself was incorrect; malinformation is more nuanced and likely to mislead precisely because the primary information may not be incorrect but selectively presented.

Lewandowsky and Cook [26] proposed a tactic for combatting conspiratorial thinking, misinformation, and disinformation that they characterize as “inoculation” or “prebunking,” with two distinct aspects: (1) an “explicit warning of an impending threat of being misled” and (2) “refutation of the misinformation’s arguments,” noting that fact- and logic-based inoculations have been successful in prebunking a 9/11 conspiracy theory [27]. Malinformation is more difficult to inoculate against because it is more difficult to predict how legitimate stories, studies, and information will be weaponized as malinformation than it is to delineate the general characteristics of misinformation and disinformation, particularly when based on conspiratorial thinking. However, we would counter that it is useful to publicly document instances of malinformation to elucidate why they constitute malinformation, as once articulated it can be applied to other stories of this sort.

How journals contribute to malinformation remains poorly understood, but editors need to be aware of how legitimate (or seemingly legitimate) stories and studies can be turned into malinformation. Accordingly, we suggest that in the age of social media, it is imperative that peer review, editorial oversight, and fact-checking go beyond just the issue of whether individual facts and findings reported in a paper are accurate and consider the overall context (or lack thereof) in which the “facts” are arranged to produce a narrative, a point expanded upon in Multimedia Appendix 1. There is increasing recognition that much of what is published in medical journals may not be reproducible and that biased, or outright fabricated results, taint biomedical literature [28-30], an alarmingly common occurrence [31]. Inevitably, dangerous falsehoods and polemics can sometimes appear in literature. It is vital that reviewers and editors are aware of these issues and skeptically evaluate potentially dubious claims. While some guidelines exist [32], wider awareness is urgently required.

In the pre–social media era when medical journals were read predominantly by clinicians and scientists, the failure of peer reviewers and editors to identify malinformation like this story would likely have had only a minor impact on the public perception of vaccine safety and efficacy, with studies like Andrew Wakefield’s fraudulent research in *The Lancet* being very exceptional cases that reduced vaccine confidence [23,33]. Unfortunately, the situation has changed markedly in the social media era. Now, the inability on the part of peer reviewers and editors to recognize malinformation before it is published under the imprimatur of a respected medical journal can have major repercussions because, as demonstrated in this analysis, such malinformation can be amplified widely.

It is crucial that scientific publications strive to communicate vital findings while also being aware that the audience for scientific work now extends far beyond the confines of academia. It is thus inevitable that highly uncertain or nuanced findings in medicoscientific journals will be stripped of context by malicious actors seeking to undermine public health and understanding. While findings can and should be debated by scientists, it is very naive to ignore the reality that these specialized debates will also transpire in the public sphere, driven by the characteristic context collapse of social media. This new normal has ramifications for how scientific publishers and scientists frame their work and their responsibilities after publication. Journals and authors need also be mindful of how their work is interpreted after publication, including issuing clarifications and correctives to address situations in which their publications are weaponized as malinformation.

Potentially controversial findings, when they arise, should not be censored, but rigorously evaluated and reported in proper context, ideally including independent lay summaries to stave off potential distortion in the public arena. If journal editors and peer reviewers are not trained to recognize potential malinformation when they see it, peer-reviewed biomedical journals can all too easily inadvertently become powerful sources and amplifiers of malinformation, rather than what they should be—bulwarks against it.

### Limitations

While our analysis showed that antivaccine sources were most likely to propagate the offending piece, it was not possible to quantify the proportion of receptive audiences who already had vaccine-negative attitudes and how much it might remain within an already committed echo chamber. Even so, there is already ample evidence that mere exposure to antivaccine sentiment induces vaccine hesitancy [6]. The volume of vaccine disinformation available online and offline creates a lingering public perception that vaccines might be harmful due to the psychological phenomena of the illusory truth effect, the observation that a statement is more likely to be deemed true after repeated exposure, regardless of intrinsic veracity [34]. As fiction is spread more efficiently across social media than corrective information [35], it is reasonable to conclude that the high publicity this piece garnered, reflected in its staggering altmetric score, was likely to induce novel vaccine hesitancy and undermine public health.

There is also some unavoidable ambiguity in defining the precise border of what constitutes malinformation. In scientific publications, erroneous claims that are perpetuated under usual circumstances might be deemed misinformation. However, as outlined in the potential mechanisms of malinformation in this work, the marshaling of claims produces a misleading and harmful picture without adequate context and is typically better described as malinformation. Moreover, even completely accurate works can be weaponized by vested interests and used as malinformation, despite no error or intention on the authors’ part. There can also be synergy between the 2 mechanisms; dubious claims can be adopted and championed by bad-faith actors, amplifying harmful effects. The results of this analysis suggest that this facet may have been evident in this instance, but it is important to recognize that such distinctions will be context-specific in many cases.

There have been many hundreds of papers on COVID-19 misinformation and disinformation since the pandemic began, and only a small number have been concentrated on altmetrics of misinformation, tracing, for example, engagement with retracted papers [36]. Potential sources of confusion with the altmetric data have included in particular numerous changes to Twitter (now X) since 2022 that have reduced transparency and an exodus of users, which could have caused altmetrics to change with time. This analysis circumvents these issues by virtue of having been performed prior to these drastic access changes, with tweets included (Multimedia Appendix 1), but will likely make future approaches using this methodology limited in scope. The use of manual sentiment sorting was time-intensive, and future iterations might take a natural language processing approach or use large language model–based artificial intelligence, but the by-hand verification of the relatively small number of high engagement tweets on the subject combined with the analysis of users’ prior vaccine positions should make conclusion in this work relatively robust.

### Conclusions

Vaccine malinformation was far more likely to be shared with negative editorialization by vaccine-negative accounts and repeatedly cited by media sources opposed to vaccination. This work highlights how malinformation is an issue that should be considered in scientific communication in the social media era to avoid the propagation of misleading and harmful narratives.

## Appendix

Multimedia Appendix 1 Supplementary material and redacted tweets.

## Data Availability

All raw tweet data analyzed during this study are included in this published paper and its supplementary information files in redacted form. The altmetric dataset and full tweet history analyzed and the raw code generated during this study are not publicly available due to potentially identifying information but are available from the corresponding author on reasonable request.
