# Supplementary material for: Quantifying Public Engagement With Science and Malinformation on COVID-19 Vaccines: Cross-Sectional Study
Source: J Med Internet Res. 2025 Mar 21;27:e64679. doi: 10.2196/64679 (PMC11971574; doi:10.2196/64679)
Supplement: Multimedia Appendix 1 [file jmir_v27i1e64679_app1.pdf]

Malinformation – an emergent problem for medical journals and scientific communication

Electronic supplemental material

David Robert Grimes[1,2*] and David H. Gorski[3,4*]

Affiliations:

1. Department of Biomedical physics, Dublin City University, Glasnevin, Dublin 9; davidrobert.grimes@dcu.ie
2. School of radiation therapy, Trinity College Dublin, St James Hospital campus, Dublin 8, Dublin, Ireland
3. Michael and Marian Ilitch Department of Surgery, Wayne State University School of Medicine, Detroit, MI, USA; gorskid@med.wayne.edu
4. Barbara Ann Karmanos Cancer Institute, Detroit, MI, USA.

**Both authors contributed equally to this work*

Gini co-efficient calculations and implementation

The Gini co-efficient for alt-metrics can be calculated by interrogation of the derived Lorenz curve. Briefly, this was calculated by sorting the raw altmetric data for all articles between April 2012 and April 2022 in five leading medical journals (New England Journal of Medicine, The Lancet, JAMA, Annals of Internal Medicine, and British Medical Journal), included in the supplementary comma separated variable file "*5medicaljournalsallimpact.csv*". This yielded n = 84,529 articles, which were sorted by altmetric score (AS), yielding a highly skewed distribution with minimum AS = 0, maximum AS = 45,866, median AS = 16 and mode AS = 1.

The Lorenz curve was calculated by creating step-sizes of $\frac{1}{n}$ and calculating the area under the curve using trapezoidal numerical integration, *A*. Under perfect equality, the area under the Lorenz curve is the area of a unit triangle, equal to 0.5. The Gini co-efficient is the difference between this theoretical area and the area under the Lorenz curve, and can be calculated from this by exploiting trigonometric identities to yield

$$G = 1 - 2B.$$

High engagement tweets

A complete collection of all tweets on the article with over 50 engagements (likes) is given in the following pages. Tweets with strike throughs were excluded from the analysis as they did not refer to the paper in question. The preexisting sentiment of all users involved was also calculated by extracting publicly available records. This data is available upon request.

Created by DRG792 Powered by wakelet

BMJ Pfizer tweets

97 Items

pfizer bmj (whistle OR blower OR fraud OR trials OR ventavia OR whistleblower) min_faves:50

Over 1000 Likes

[redacted]

"Press release: Research fraud at Pfizer subcontractor. Now 16 doctors and researchers are calling for vaccination in Sweden to be paused." lakaruppropet.se/wp-content/upl...
"The British Medical Journal (BMJ) reveals on 2 November 2021 that a subcontractor to Pfizer is likely to have...

2:43 PM · Nov 6, 2021

[redacted] Read the full conversation on Twitter

10.8K See the latest COVID-19 information on Twitter

Read 235 replies

[redacted]

BREAKING:

BMJ whistle blower investigation reveals evidence of falsifying data in pivotal Pfizer Covid vaccine trial

‘Where is the criminal FBI investigation?’

‘Drug companies paid fines of $13 billion between 2009 - 2014 and nothing has been done to rectify the problem’

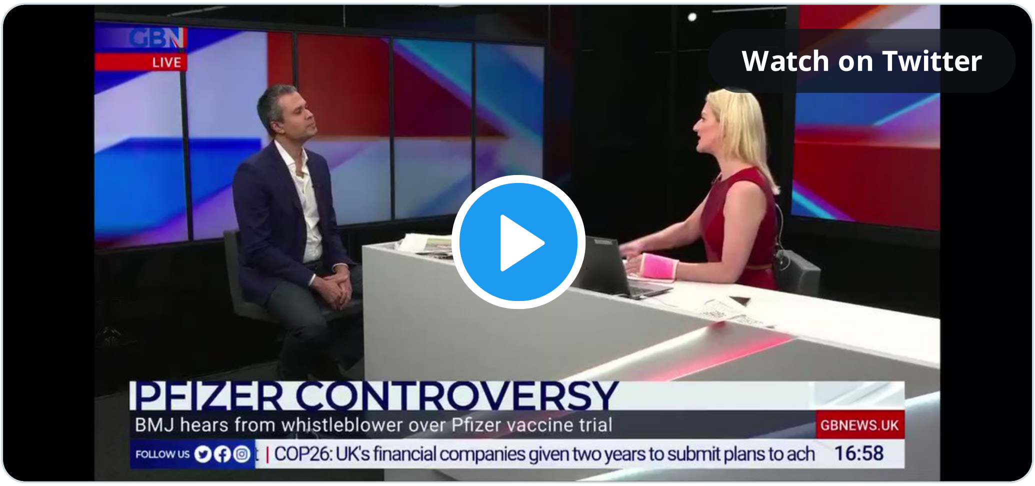

7:03 PM · Nov 4, 2021

9.7K   See the latest COVID-19 information on Twitter

Read 405 replies

The BMJ @bmj_latest · Nov 2, 2021

NEW: Our latest investigation hears from a whistleblower engaged in Pfizer's pivotal covid-19 vaccine trial. Her evidence raises serious questions about data integrity and regulatory oversight

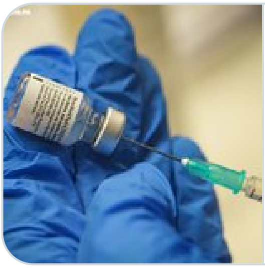

bmj.com
Covid-19: Researcher blows the whistle on...
Revelations of poor practices at a contract research company helping to carry out ...

[redacted]

Imagine needing a whistleblower to tell you what history has proven time and time again. Pfizer is a habitual offender. Corruption is in their DNA. They regularly value profits over human lives. To let any one of their products near your family is lunacy.

2:19 PM · Nov 2, 2021 from Georgia, USA

8.5K See the latest COVID-19 information on Twitter

Read 99 replies

[redacted]

💣Une fraude de grande ampleur, impliquant le laboratoire Pfizer, son sous-traitant la société Ventavia chargée des essais cliniques, et la @US_FDA, révélée hier par le British Medical Journal @bmj_latest : la nouvelle fait l'effet d'une bombe. #PfizerGate

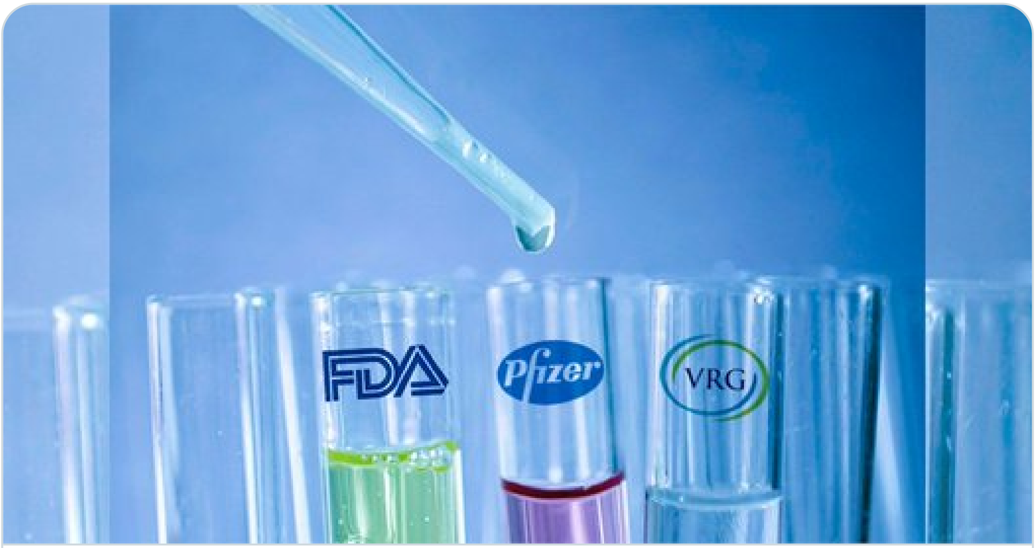

francesoir.fr
"PfizerGate" ? Révélations sur des essais cliniques falsifiés par le tript...
Une fraude de grande ampleur, impliquant la société pharmaceutique Pfizer, son sous-traitant, la société Ventavia Research Group chargée...

11:59 AM · Nov 3, 2021

4.3K See the latest COVID-19 information on Twitter

Read 175 replies

[redacted]

#PfizerGate révélé par une lanceuse d'alerte dans le BMJ ! L'ancienne cadre de Ventavia, sous-traitant de Pfizer chargé des essais cliniques, explique que les données ont été falsifiées, les patients non suivis malgré des effets indésirables, l'étude menée sans double aveugle !!!

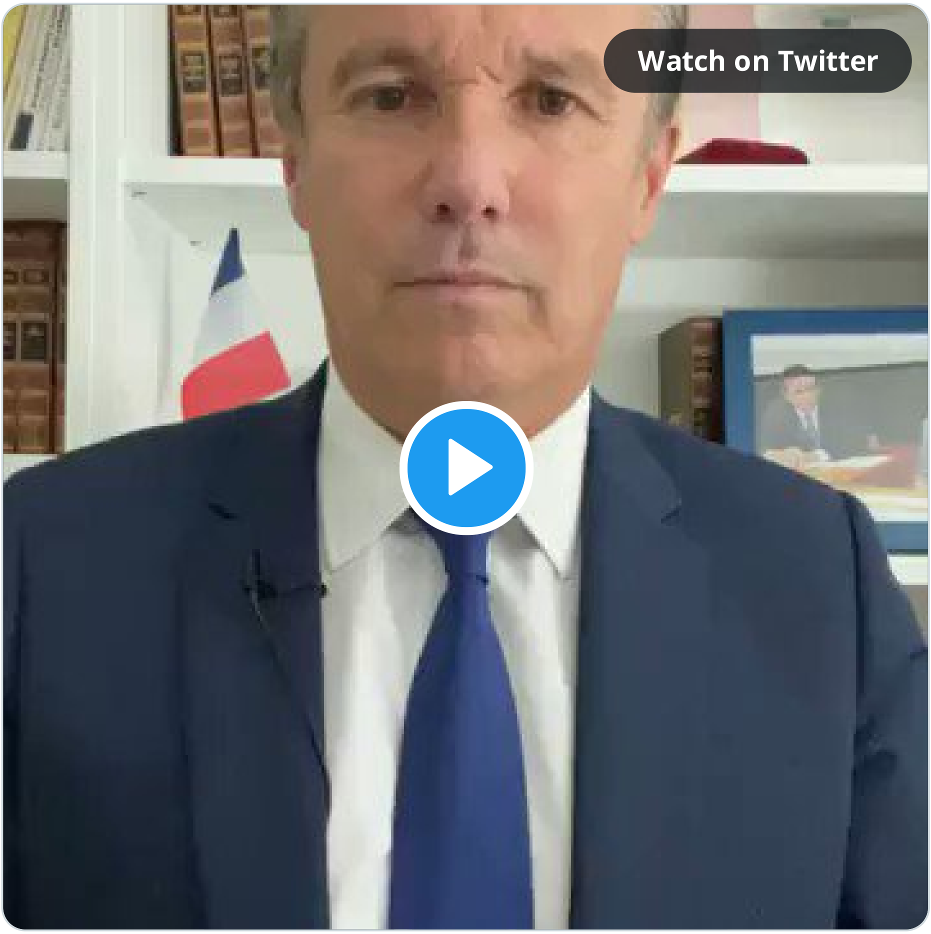

2:29 PM · Nov 3, 2021

[redacted] Read the full conversation on Twitter

3.9K See the latest COVID-19 information on Twitter

Read 293 replies

[redacted]

Is Bill Gates an enemy of democracy? If not why has LinkedIn removed my LBC interview with @MaajidNawaz on the Pfizer data whistleblower scandal exposed by the BMJ ?

youtu.be/AjHoca9wgoY

bmj.com/content/375/bm…

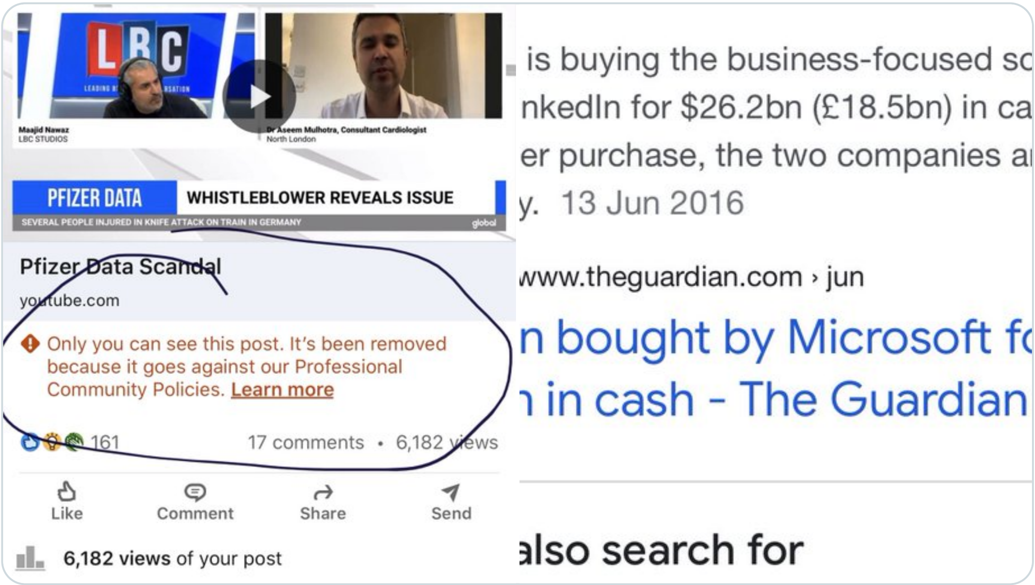

6:50 AM · Nov 8, 2021

2.9K See the latest COVID-19 information on Twitter

Read 186 replies

[redacted]

The BMJ has posted a story that calls into question the reliability of the data generated by Pfizer's vaccine trials. It's based on dozens of documents, photos, audio recordings and emails supplied by a whistleblower.

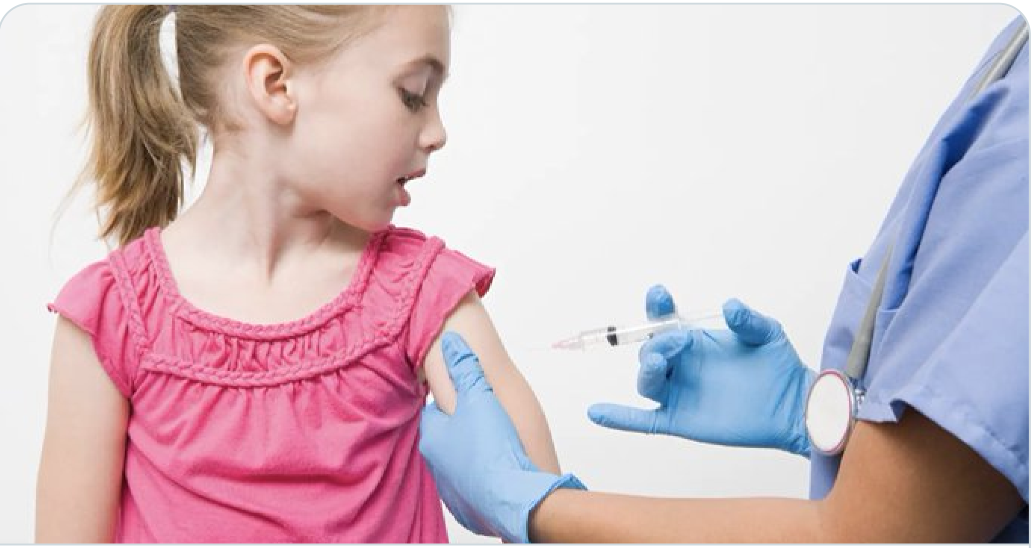

dailysceptic.org

Researcher Blows the Whistle on Data Integrity Issues in Pfizer's Vac...

The BMJ has posted a story that calls into question the reliability of the data generated by Pfizer's vaccine trials. It's based on dozens of ...

4:08 PM · Nov 2, 2021

2.4K See the latest COVID-19 information on Twitter

Read 91 replies

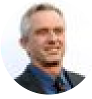

Robert F. Kennedy Jr
@RobertKennedyJr

WATCH as @jimmy_dore discusses @bmj_latest report exposing Pfizer's Phase 3 COVID vaccine trial for having fake data, blind trial failures, delayed follow-ups on serious adverse reactions and silencing of researchers who were critical of trials practices.

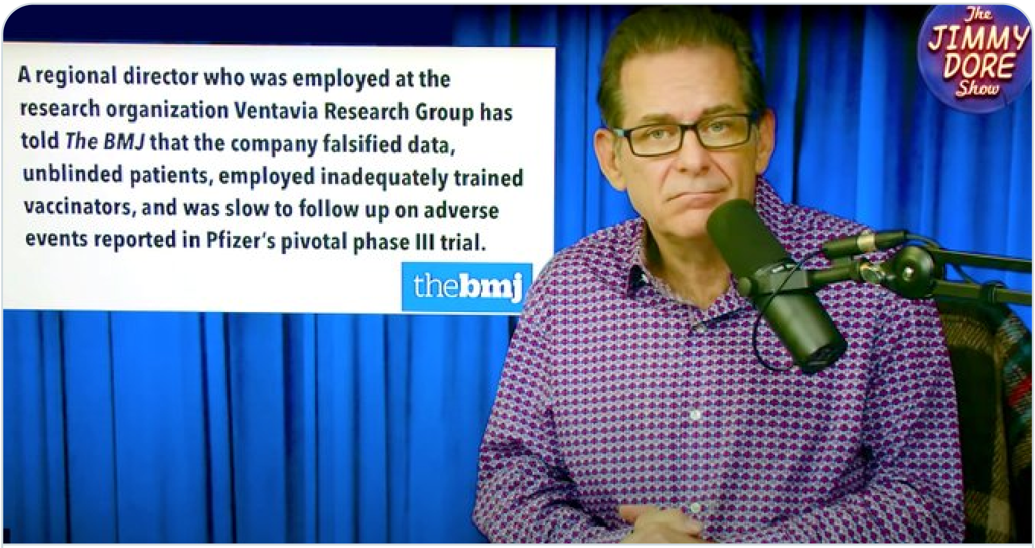

childrenshealthdefense.org
Researcher Speaks Out on Pfizer COVID Vaccine Trial, Calls It a 'Craz…
On the latest episode of "The Jimmy Dore Show," comedian and political commentator Jimmy Dore discussed a report by The BMJ o…

4:54 PM · Nov 15, 2021

1.9K   See the latest COVID-19 information on Twitter

Read 54 replies

[redacted]

So etwas untergräbt natürlich das Vertrauen in die Validität der #Pfizer Daten: „ #Covid19 Researcher blows the whistle on data integrity issues in Pfizer’s #vaccine trial“ @bmj_latest 👉 Und genau darum sind unabhängige Studien so wichtig !#STIKO

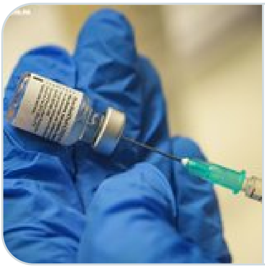

bmj.com
Covid-19: Researcher blows the whistle on...
Revelations of poor practices at a contract research company helping to carry out ...

5:23 PM · Nov 2, 2021

[redacted] Read the full conversation on Twitter

1.6K See the latest COVID-19 information on Twitter

Read 187 replies

[redacted]

Als dit waar is dan zitten wij in grote problemen. Covid-19: Researcher blows the whistle on data integrity issues in Pfizer’s vaccine trial | The BMJ

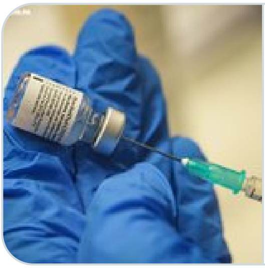

bmj.com
Covid-19: Researcher blows the whistle on data in...
Revelations of poor practices at a contract research company helping to carry out Pfizer’s ...

10:34 PM · Nov 6, 2021

1.4K See the latest COVID-19 information on Twitter

Read 637 replies

[redacted]

This is ALARMING and should make for uncomfortable reading! Covid-19: Researcher blows the whistle on data integrity issues in Pfizer's vaccine trial | The BMJ

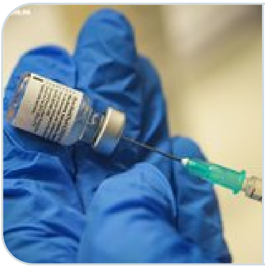

bmj.com

Covid-19: Researcher blows the whistle on data in...

Revelations of poor practices at a contract research company helping to carry out Pfizer's ...

10:37 AM · Nov 4, 2021

1.2K See the latest COVID-19 information on Twitter

Read 70 replies

[redacted]

"Researcher blows the whistle on data integrity issues in Pfizer's vaccine trial"

Published today in the BMJ, one of the top 5 medical journals in the world.

Full article:
bmj.com/content/375/bm…

thebmj

Feature

BMJ Investigation

Covid-19: Researcher blows the whistle on data integrity issues in Pfizer's vaccine trial

BMJ 2021 ; 375 doi: https://doi.org/10.1136/bmj.n2635 (Published 02 November 2021)

Cite this as: *BMJ* 2021;375:n2635

5:05 PM · Nov 2, 2021

1.1K See the latest COVID-19 information on Twitter

Read 38 replies

500-1000 likes

[redacted]

Pfizer asi denemeleriyle ilgili sonuclarinin guvenilirligindeki supheler FDA ya rapor ediliyor ve FDA da TIK yok! Bu nasil bir bilim etigiyse artik.

Covid-19: Researcher blows the whistle on data integrity issues in Pfizer’s vaccine trial | The BMJ

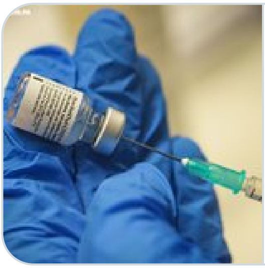

bmj.com
Covid-19: Researcher blows the whistle on...
Revelations of poor practices at a contract research company helping to carry out ...

8:26 PM · Nov 3, 2021

[redacted]

Read the full conversation on Twitter

936 See the latest COVID-19 information on Twitter

Read 19 replies

[redacted]

For me, I can't stop thinking about Pfizer's fine for $2.3 billion in 2009 for fraudulent marketing, about their previous fines for data manipulation.

I think about the BMJ whistleblower article.

And, then, I read this, misreporting the death data?

I don't trust Pfizer.

[redacted]

alexberenson.substack.com/p/more-people-...

More people died in the key clinical trial for Pfizer's Covid vaccine than the company publicly reported

Pfizer told the world 15 people who received the vaccine in its trial had died as of mid-March. Turns out the real number then was 21, compared to only 17 deaths in people who hadn't been vaccinated.

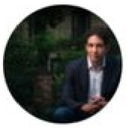

Alex Berenson

Nov 16

6:51 PM · Nov 16, 2021

690 See the latest COVID-19 information on Twitter

Read 22 replies

[redacted]

Zaczyna się. Nie chciałbym już być w skórze PiS.
--
Dyrektor regionalny, który był zatrudniony w firmie Ventavia Research Group robiącej badania preparatów dla Pfizer'a powiedział The BMJ, że firma fałszowała dane, zatrudniała niewłaściwie przeszkolonych szczepiących ...

> **The BMJ** @bmj_latest
> NEW: Our latest investigation hears from a whistleblower engaged in Pfizer's pivotal covid-19 vaccine trial. Her evidence raises serious questions about data integrity and regulatory oversight ow.ly/9RXL50GEjoT

7:54 PM · Nov 2, 2021

[redacted]

Read the full conversation on Twitter

613 See the latest COVID-19 information on Twitter

Read 19 replies

[redacted]

Mein Take der @bmj_latest-Story zu #Pfizer: Vertrauen erschüttert. Und vor allem: Angesichts des von @thackerpd geschilderten Umgangs mit den 1000 #Ventavia-Probanden sollte dringend ermittelt werden, wie bei den restlichen 39.000 Versuchspersonen gearbeitet wurde.

9:54 AM · Nov 4, 2021

575 See the latest COVID-19 information on Twitter

Read 35 replies

Sputnik V
@sputnikvaccine

The amazing power of Pfizer to silence Western media illustrated in two breaking stories of today. No additional reporting on major articles published in respected BMJ and Nature. Evident double standards and complete radio silence of Western media on:

7:46 PM · Nov 2, 2021

Read the full conversation on Twitter

549 See the latest COVID-19 information on Twitter

Read 32 replies

[redacted]

The BMJ published an investigation of Pfizer's vaccine clinical trial bmj.com/content/375/bm… Ventavia is falsely claiming Brook Jackson never worked on the clinical trial. Her lawyer sent Ventavia a cease & desist for defamation documentcloud.org/documents/2111… /1

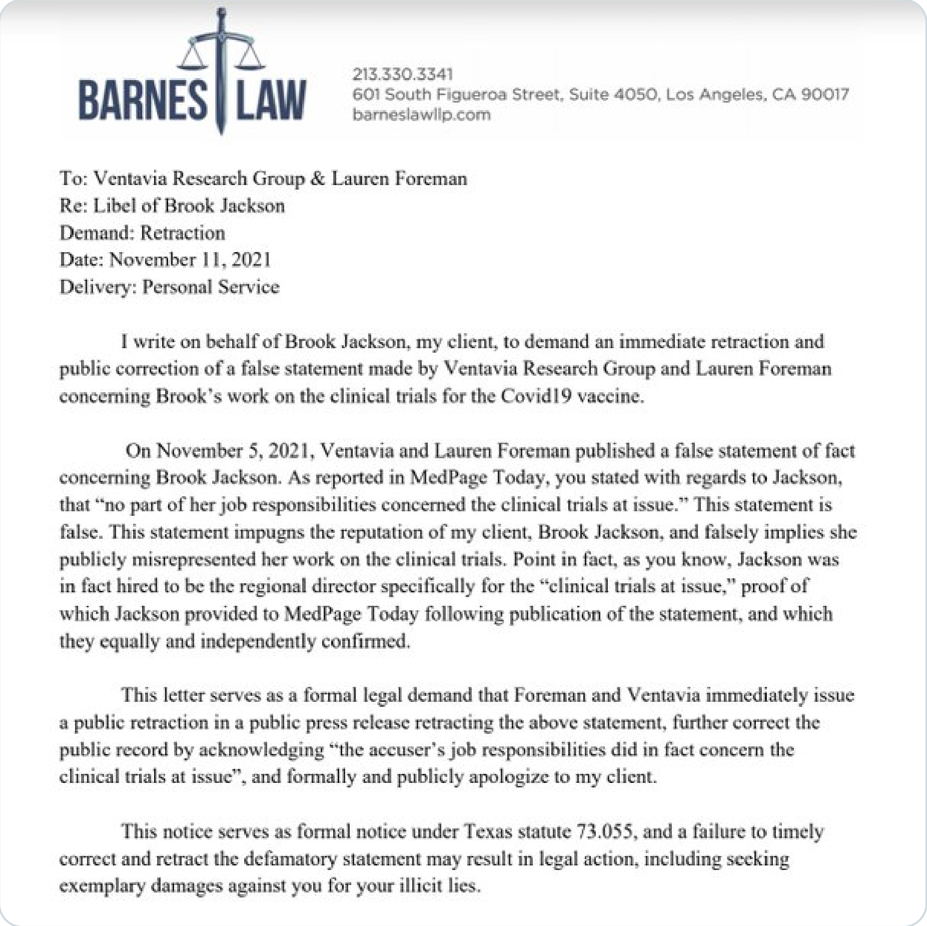

BARNES LAW

213.330.3341
601 South Figueroa Street, Suite 4050, Los Angeles, CA 90017
barneslawllp.com

To: Ventavia Research Group & Lauren Foreman
Re: Libel of Brook Jackson
Demand: Retraction
Date: November 11, 2021
Delivery: Personal Service

I write on behalf of Brook Jackson, my client, to demand an immediate retraction and public correction of a false statement made by Ventavia Research Group and Lauren Foreman concerning Brook's work on the clinical trials for the Covid19 vaccine.

On November 5, 2021, Ventavia and Lauren Foreman published a false statement of fact concerning Brook Jackson. As reported in MedPage Today, you stated with regards to Jackson, that "no part of her job responsibilities concerned the clinical trials at issue." This statement is false. This statement impugns the reputation of my client, Brook Jackson, and falsely implies she publicly misrepresented her work on the clinical trials. Point in fact, as you know, Jackson was in fact hired to be the regional director specifically for the "clinical trials at issue," proof of which Jackson provided to MedPage Today following publication of the statement, and which they equally and independently confirmed.

This letter serves as a formal legal demand that Foreman and Ventavia immediately issue a public retraction in a public press release retracting the above statement, further correct the public record by acknowledging "the accuser's job responsibilities did in fact concern the clinical trials at issue", and formally and publicly apologize to my client.

This notice serves as formal notice under Texas statute 73.055, and a failure to timely correct and retract the defamatory statement may result in legal action, including seeking exemplary damages against you for your illicit lies.

2:24 PM · Nov 18, 2021

[redacted]

Read the full conversation on Twitter

457 See the latest COVID-19 information on Twitter

Read 12 replies

[redacted]

New Pfizer whistleblower tells "The BMJ" that the company falsified data, among other violations of research protocols.

HUGE.

bmj.com/content/375/bm...

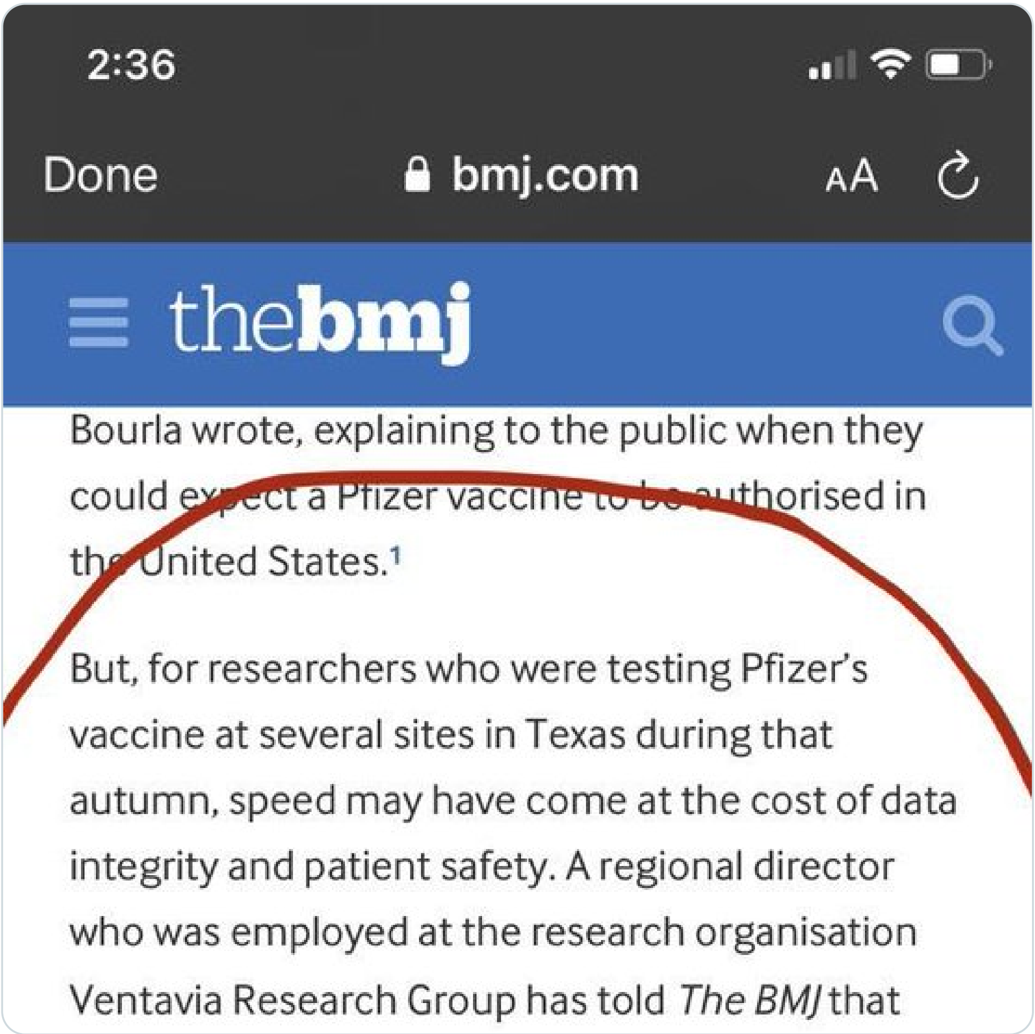

Bourla wrote, explaining to the public when they could expect a Pfizer vaccine to be authorised in the United States.[1]

But, for researchers who were testing Pfizer's vaccine at several sites in Texas during that autumn, speed may have come at the cost of data integrity and patient safety. A regional director who was employed at the research organisation Ventavia Research Group has told *The BMJ* that

9:38 PM · Nov 2, 2021

454 See the latest COVID-19 information on Twitter

Read 7 replies

[redacted]

Shame on BMJ for irresponsible reporting.

The serious data problems found with Ventavia's management of Pfizer's vaccine trial:

likely make the vaccine look worse than it actually is

affect 3 of 153 sites

You need to lead with this information, @bmj_latest. So disappointing.

> **The BMJ** @bmj_latest
> NEW: Our latest investigation hears from a whistleblower engaged in Pfizer’s pivotal covid-19 vaccine trial. Her evidence raises serious questions about data integrity and regulatory oversight ow.ly/9RXL50GEjoT

5:25 PM · Nov 3, 2021

443   See the latest COVID-19 information on Twitter

Read 44 replies

[redacted]

Cet article est la traduction littérale du rapport de Paul Thacker paru le 2 novembre dans British Médical Journal (BMJ) concernant les graves manquements du laboratoire Ventavia chargé de réaliser une partie des essais de phase III du vaccin Pfizer.

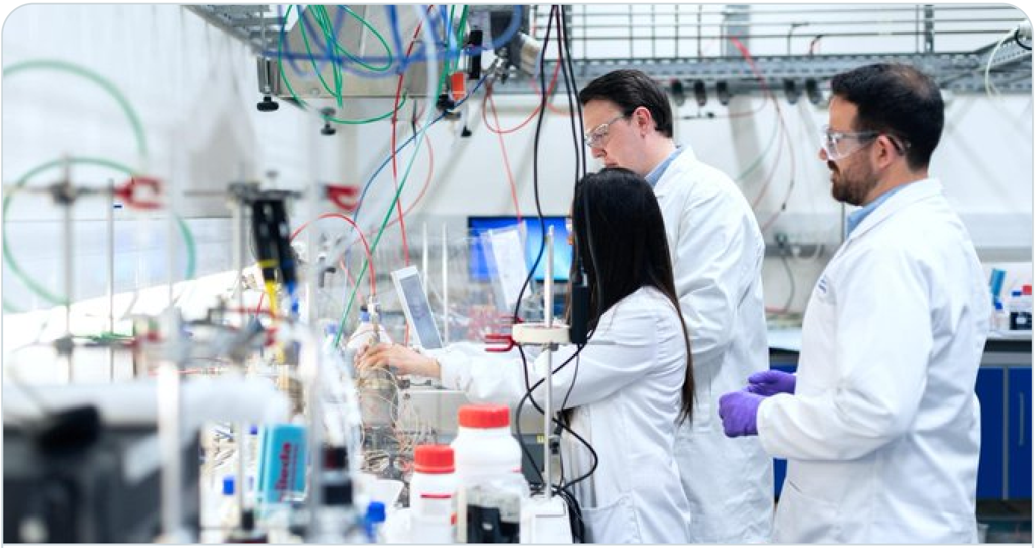

reinfocovid.fr
COVID-19 : Un chercheur dénonce des problèmes d'intégrité des do...
Cet article est la traduction littérale du rapport de Paul Thacker paru le 2 novembre dans British Médical Journal (BMJ) concernant les ...

10:05 AM · Dec 5, 2021

434 See the latest COVID-19 information on Twitter

Read 4 replies

[redacted] v 4, 2021

Sulla questione #Pfizergate, sono necessarie alcune considerazioni: small 3D
1/n

[redacted]

Il BMJ pubblica le dichiarazioni di un whistleblower (dipendente di un azienda che ne denuncia in maniera anonima le pratiche scorrette) che denuncia Pfizer per una serie di scorrettezze e irregolarità.
Il whistleblower (WB) non lavora per Pfizer ma per Ventavia.
2/n

» BMJ Investigation

9: Researcher blows the whistle on data integrity issues in Pfizer's v[illegible]

; 375 doi: https://doi.org/10.1136/bmj.n2635 (Published 02 November 2021)
[illegible]: *BMJ* 2021;375:n2635

[illegible]ur latest coverage of the coronavirus pandemic

Related content | Metrics | Responses

9:23 AM · Nov 4, 2021

415 See the latest COVID-19 information on Twitter

Read 19 replies

[redacted]

In the BMJ today....
Covid-19: Researcher blows the whistle on data integrity issues in Pfizer's vaccine trial
bmj.com/content/375/bm...

2:40 PM · Nov 2, 2021

[redacted] Read the full conversation on Twitter

392 See the latest COVID-19 information on Twitter

Read 12 replies

[redacted]

BMJ hears from Pfizer Whistleblower about alleged falsification of test data and failure to follow up vaccine injuries!

Dr Aseem Malhotra @DrAseemMalhotra

Pfizer whistleblower scandal - why vaccine mandates are unscientific and unethical

'If you mandate a vaccine for #NHS staff on non transparent data you'll only widen the chasm of distrust'

We'll also risk losing 100k staff & THAT will be devastating

youtu.be/m1c-oPF38FY

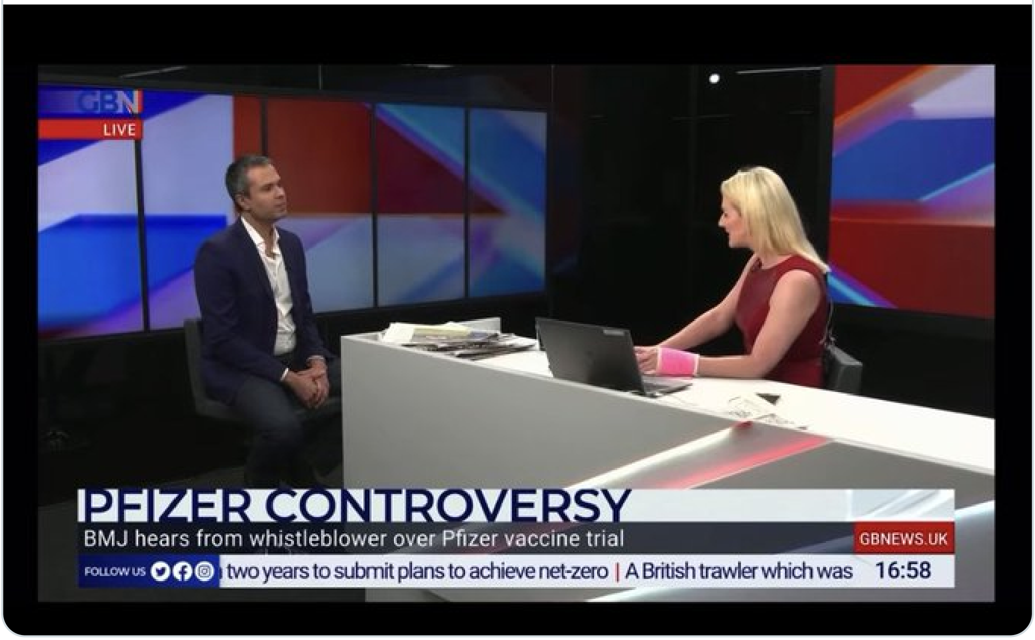

10:54 PM · Dec 14, 2021

335 See the latest COVID-19 information on Twitter

Read 4 replies

[redacted]

.@paulfrewDUP re @bmj_latest Pfizer whistleblower incident.

"I believe that the Health Minister should make a statement to this house on what action he can take to assuage the concerns of people that have taken the Pfizer vaccine."

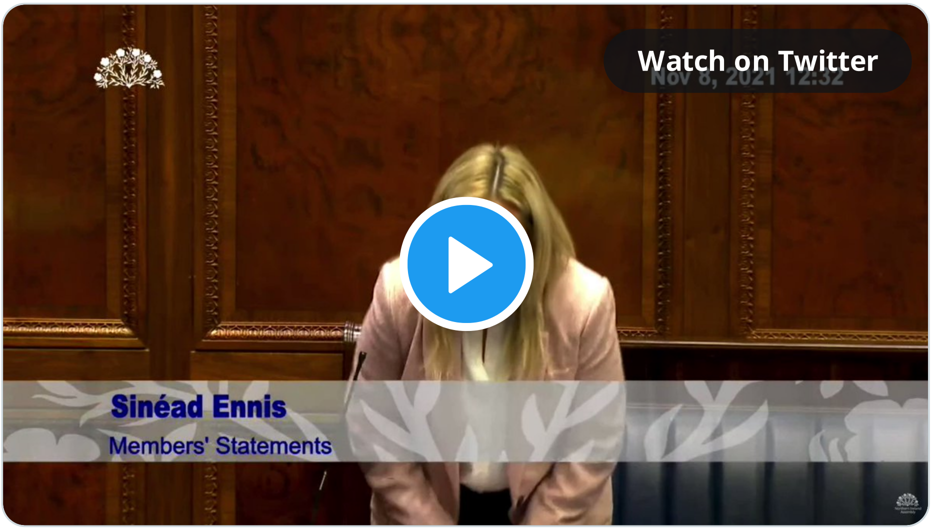

4:47 PM · Nov 8, 2021

[redacted]

Read the full conversation on Twitter

334 See the latest COVID-19 information on Twitter

Read 19 replies

[redacted]

ファイザー不正疑惑の内部告発をＢＭＪが報じました。

Covid-19: Researcher blows the whistle on data integrity issues in Pfizer’s vaccine trial

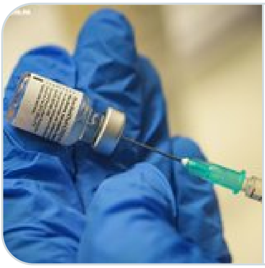

bmj.com
Covid-19: Researcher blows the whistle on data in...
Revelations of poor practices at a contract research company helping to carry out Pfizer’s ...

2:04 AM · Nov 4, 2021

331 See the latest COVID-19 information on Twitter

Read 14 replies

[redacted]

NEW: Our latest investigation hears from a whistleblower engaged in Pfizer’s pivotal covid-19 vaccine trial. Her evidence raises serious questions about data integrity and regulatory oversight. @bmj_latest @thackerpd

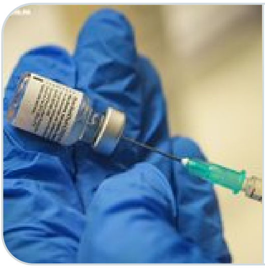

bmj.com
Covid-19: Researcher blows the whistle on data in...
Revelations of poor practices at a contract research company helping to carry out Pfizer’s ...

2:21 PM · Nov 2, 2021

328 See the latest COVID-19 information on Twitter

Read 16 replies

[redacted]

Covid-19: A new @bmj_latest investigation, informed by a whistleblower, reveals how @FDA failed to investigate a series of alarming data integrity irregularities during the Pfizer vaccine trial

@thackerpd with the story

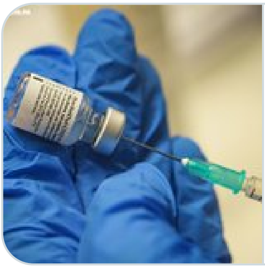

bmj.com
Covid-19: Researcher blows the whistle on data in...
Revelations of poor practices at a contract research company helping to carry out Pfizer's ...

3:55 PM · Nov 2, 2021

309 See the latest COVID-19 information on Twitter

Read 19 replies

[redacted]

Pfizer falsified data, broke fundamental rules, and were 'slow' to report adverse reactions.
Covid-19: Researcher blows the whistle on data integrity issues in #Pfizer's vaccine trial | The BMJ @bmj_latest @pfizer

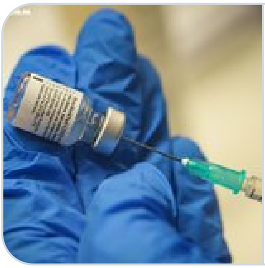

bmj.com
Covid-19: Researcher blows the whistle on data in...
Revelations of poor practices at a contract research company helping to carry out Pfizer's ...

2:32 AM · Nov 3, 2021

308 See the latest COVID-19 information on Twitter

Read 13 replies

[redacted]

Pfizer adına covid aşısıyla ilgili saha araştırmaları yapan Ventavia Araştırma Örgütü'nün bölge müdürü Brook Jackson, FDA'ya bir şikayet e-postası gönderdi. Ventavia aynı gün Jackson'u kovdu.

Skandalı, saygın tıp dergisi @bmj_latest Dünya'ya duyurdu.

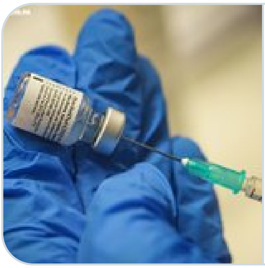

bmj.com
Covid-19: Researcher blows the whistle on...
Revelations of poor practices at a contract research company helping to carry out ...

4:47 PM · Nov 5, 2021

[redacted] Read the full conversation on Twitter

299 See the latest COVID-19 information on Twitter

Read 2 replies

[redacted]

Väärinkäytöksiä Pfizerin tutkimuksissa. Koskettaa aika montaa ihmistä maapallolla, mutta mediat ovat niin hiljaa että korviin sattuu.

Covid-19: Researcher blows the whistle on data integrity issues in Pfizer’s vaccine trial | The BMJ

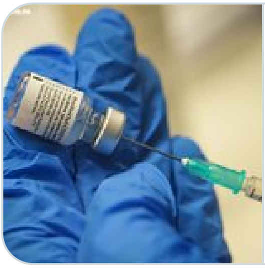

bmj.com
Covid-19: Researcher blows the whistle on data in...
Revelations of poor practices at a contract research company helping to carry out Pfizer’s ...

7:55 AM · Nov 7, 2021

293 See the latest COVID-19 information on Twitter

Read 6 replies

The BMJ
@bmj_latest

NEW: An investigation published by @bmj_latest this week hears from a whistleblower engaged in Pfizer’s pivotal covid-19 vaccine trial. Her evidence raises serious questions about data integrity and regulatory oversight

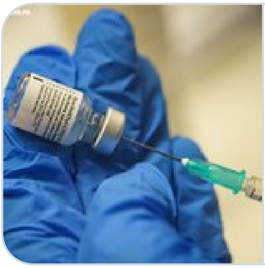

bmj.com
Covid-19: Researcher blows the whistle on data in...
Revelations of poor practices at a contract research company helping to carry out Pfizer’s ...

11:00 AM · Nov 6, 2021

287 See the latest COVID-19 information on Twitter

Read 19 replies

[redacted]

1) “Um diretor regional que trabalhava na organização de pesquisa Ventavia Research Group disse ao BMJ que a empresa falsificou dados, removeu o “cegamento” de pacientes, empregou vacinadores inadequadamente treinados e demorou a acompanhar os eventos adversos relatados no estudo

[redacted]

3) enviou uma reclamação por e-mail ao US Food and Drug Administration (FDA). Ventavia a despediu mais tarde no mesmo dia. Jackson forneceu ao BMJ dezenas de documentos internos da empresa, fotos, gravações de áudio e e-mails.” - sobre o estudo da Pfizer que liberou o uso desta

10:51 PM · Nov 2, 2021

266 See the latest COVID-19 information on Twitter

Explore what's happening on Twitter

[redacted]

Se confirman las malas prácticas, falseamiento de datos de Pfizer, así como la laxa supervisión de la FDA tras haber sido avisados por trabajadores que fueron despedidos. Contrataron una empresa externa: ventavia. Y usted pagará su infamia. The BMJ. bmj.com/content/375/bm...

11:31 AM · Nov 3, 2021

[redacted]

Read the full conversation on Twitter

255 See the latest COVID-19 information on Twitter

Read 8 replies

[redacted]

Was the Pfizer CEO arrested?
Did the BMJ publish a whistleblower account of dodgy practices during the trials?
Did Peter Doshi of the BMJ just say that the claims made by Pfizer about their vaccine just don't compute?
Did Donnelly frantically shout down Veronica?

7:21 PM · Nov 5, 2021

236 See the latest COVID-19 information on Twitter

Read 30 replies

[redacted]

Highly recommend this BMJ piece by Dr. Doshi.

Explores what the trial endpts could/couldn't tell us

Pfizer pop'd it's trials with young & healthy ppl. Not those who needed treatment the most, the older and comorbid

There were only 12 ppl 65+ in the Pfizer booster trial

🧵

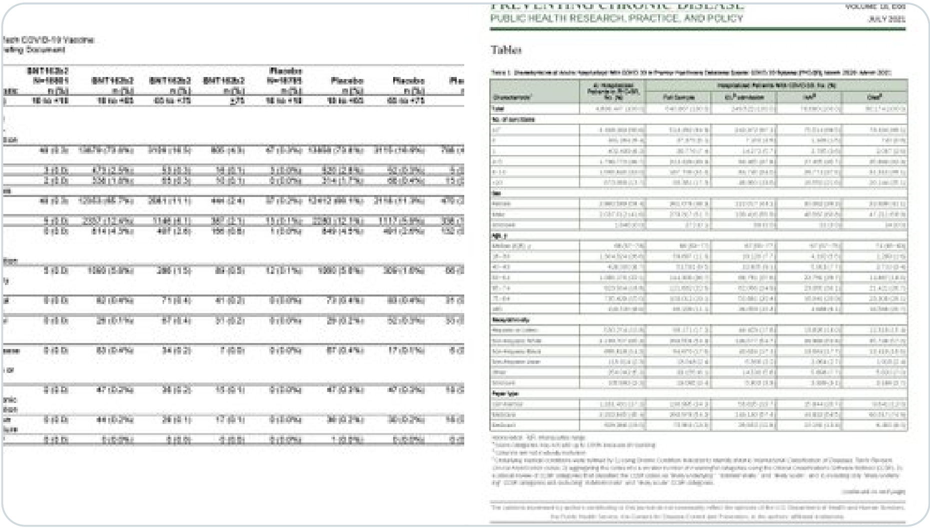

10:40 PM · Nov 22, 2021

Read the full conversation on Twitter

227 See the latest COVID-19 information on Twitter

Read 13 replies

[redacted]

"A regional director who was employed at the research org Ventavia Research Group has told The BMJ that the company falsified data, unblinded patients, employed inadequately trained vaccinator & was slow to follow up on adverse events reported in Pfizer's pivotal phase III trial"

3:07 PM · Nov 2, 2021

[redacted]

Read the full conversation on Twitter

218 See the latest COVID-19 information on Twitter

Read 5 replies

[redacted]

💣Un fraude a gran escala, que involucra al laboratorio Pfizer, su subcontratista la empresa Ventavia a cargo de los ensayos clínicos, y la @US_FDA, revelado ayer por el British Medical Journal @bmj_latest: la noticia tiene el efecto de una bomba. #PfizerGate

> **The BMJ** @bmj_latest
> NEW: Our latest investigation hears from a whistleblower engaged in Pfizer's pivotal covid-19 vaccine trial. Her evidence raises serious questions about data integrity and regulatory oversight ow.ly/9RXL50GEjoT

12:26 PM · Nov 3, 2021

218 See the latest COVID-19 information on Twitter

Read 8 replies

[redacted]

[redacted] Replying to @bmj_latest

Ok, someone alleges that a site (which handled 4% of the subjects) *may* have had some paperwork left out that could *possibly* unblind the investigators for some subjects. And you're running with THAT headline?

[redacted]

Entire trials have been tossed for less. And what gets lost in all of this, the director of the CDER who approves drug authorizations, Patrizia Cavazzoni, came to the FDA in 2018 from Pfizer. She was Sr VP of Global Development, and headed a cooperative working on mRNA platforms

6:48 PM · Nov 2, 2021

218 See the latest COVID-19 information on Twitter

Explore what's happening on Twitter

[redacted]

One of the vaccinators on the Pfizer trial had no prior medical experience at all. Her previous job was in a taco restaurant.

I wish I was making that up, but this is what verified whistleblower @IamBrookJackson, vetted by @bmj_latest, told me last week

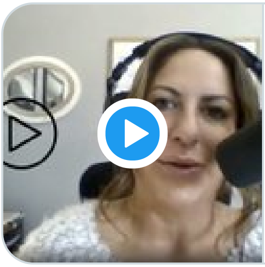

philharper.substack.com
A vaccinator on the Pfizer trial had no medical ex...
Her prior experience was working at a taco restaurant

1:59 PM · Mar 15, 2022

213 See the latest COVID-19 information on Twitter

Read 18 replies

[redacted]

+++ EIL +++ Pfizer fälschte Daten in Zulassungsstudie

Ein Whistleblower hat der Fachzeitschrift British Medical Journal (BMJ) berichtet, dass das Pharmaunternehmen Pfizer während der Zulassungsstudie seines Corona-Impfstoffs Daten fälschte

9:19 PM · Nov 3, 2021

211 See the latest COVID-19 information on Twitter

Read 6 replies

[redacted]

Why are none of these news organisations reporting the BMJ findings about Pfizer trials?

@BBCNews @itvnews @SkyNews @WalesOnline @BBCWalesNews @ITVWales

Here is the article - other counties are reporting this as you can see in my next tweet

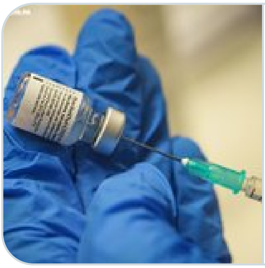

bmj.com
Covid-19: Researcher blows the whistle on...
Revelations of poor practices at a contract research company helping to carry out ...

11:57 AM · Nov 10, 2021

[redacted] Read the full conversation on Twitter

205 See the latest COVID-19 information on Twitter

Read 16 replies

[redacted]

Yesterday's BMJ story about Ventavia, a subcontractor lab for Pfizer's COVID19 vaccine trial, was misleading. It was about problems at a specific lab, which were bad, but it was presented as if all vaccine trials can not be trusted.
#VaccinesWork

[redacted]

Shame on BMJ for irresponsible reporting.

The serious data problems found with Ventavia's management of Pfizer's vaccine trial:

likely make the vaccine look worse than it actually is

affect 3 of 153 sites

You need to lead with this information, @bmj_latest.
So disappointing. twitter.com/bmj_latest/sta...

6:21 PM · Nov 3, 2021

193 See the latest COVID-19 information on Twitter

Read 34 replies

[redacted]

Good summary of the Pfizer vaccine trials and all the efforts they have made to bypass safety procedures and intentionally misreport their efficacy. By BMJ editor Peter Doshi - fast becoming the bravest man in pharma.

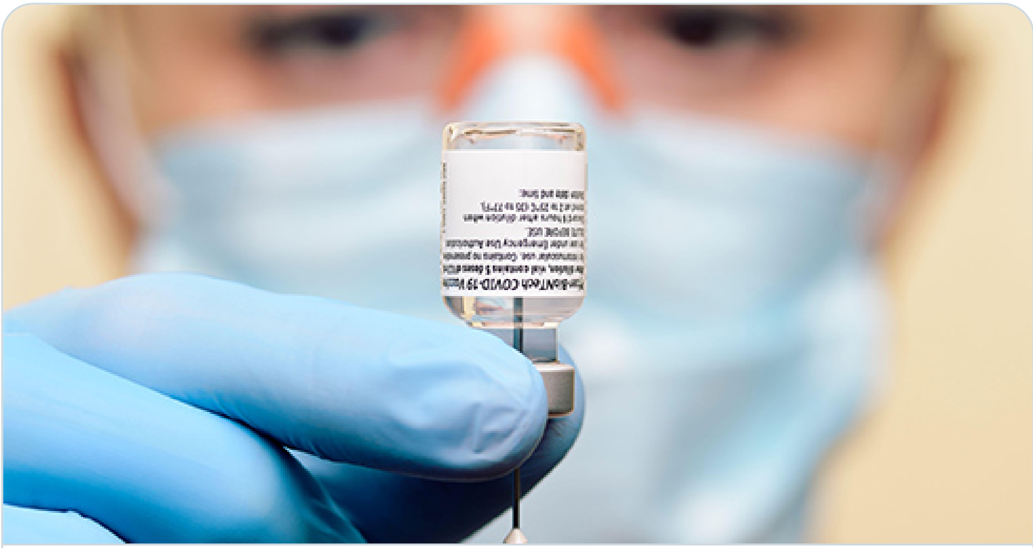

blogs.bmj.com

Does the FDA think these data justify the first full approval of a covi...

The FDA should demand adequate, controlled studies with long term follow up, and make data publicly available, before granting full ...

2:59 PM · Nov 11, 2021

189 See the latest COVID-19 information on Twitter

Read 8 replies

[redacted]

1/n Je viens de parcourir les révélations du BMJ sur Pfizer et son sous traitant Ventavia et je ne vois rien qui soit de nature à remettre en cause l'efficacité et la sécurité des vaccins.

2:32 PM · Nov 3, 2021

[redacted]

Read the full conversation on Twitter

174 See the latest COVID-19 information on Twitter

Read 37 replies

[redacted]

Australia's medicines regulator requests information from Pfizer after @bmj_latest investigation raises data integrity concerns in vaccine safety trials bit.ly/304PWOX @newscomauHQ

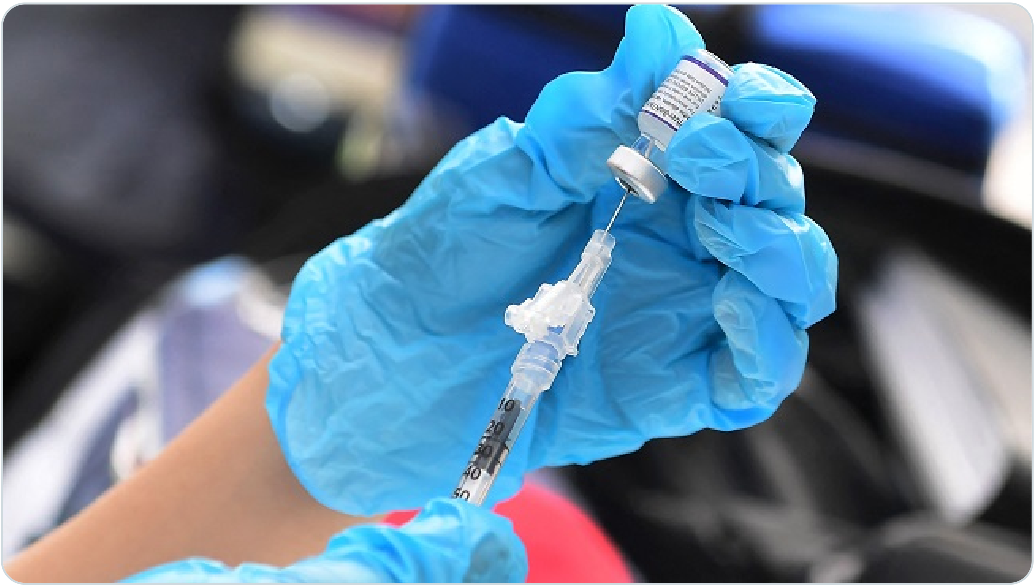

9:39 PM · Nov 9, 2021

164 See the latest COVID-19 information on Twitter

Read 34 replies

[redacted]

#Pfizergate Le plus grave dans les révélations de @bmj_latest c'est que malgré les alertes sur le sous-traitant #Ventavia, Pfizer a continué à les solliciter pour les essais d'évaluation chez les jeunes, femmes enceintes, 3e dose...
INACCEPTABLE !
#Pfizer

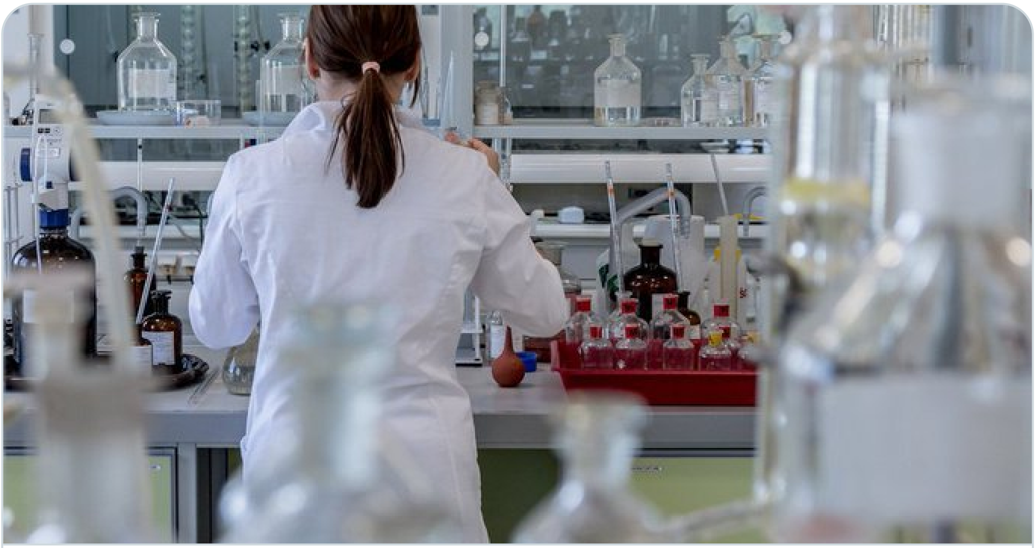

lindependant.fr
Vaccin anti-Covid - Un des sous-traitants de Pfizer dans le collimateu...
Ventavia, un des très nombreux sous-traitants de Pfizer dans la conduite des essais sur le vaccin anti-Covid, se serait montré négligen...

2:05 PM · Nov 3, 2021

162 See the latest COVID-19 information on Twitter

Read 6 replies

[redacted]

#PfizerPfusch #Medienversagen

Eine Whistleblowerin berichtete im BMJ vor einer Woche über massive Unregelmäßigkeiten bei einem Pfizer-Subunternehmen, das an den Trials beteiligt war.

Wie gestaltete sich das Echo in deutschen Medien?

Minithread
/1

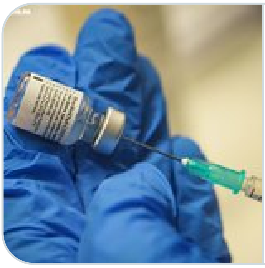

bmj.com
Covid-19: Researcher blows the whistle on...
Revelations of poor practices at a contract research company helping to carry out ...

10:47 AM · Nov 11, 2021

[redacted]

Read the full conversation on Twitter

162 See the latest COVID-19 information on Twitter

Read 9 replies

Sputnik V @sputnikvaccine · Nov 2, 2021

The amazing power of Pfizer to silence Western media illustrated in two breaking stories of today. No additional reporting on major articles published in respected BMJ and Nature. Evident double standards and complete radio silence of Western media on:

Sputnik V
@sputnikvaccine

1) British Medical Journal (BMJ) investigation: “Researcher blows the whistle on data integrity issues in Pfizer’s vaccine trial”.

> **The BMJ** @bmj_latest
> NEW: Our latest investigation hears from a whistleblower engaged in Pfizer’s pivotal covid-19 vaccine trial. Her evidence raises serious questions about data integrity and regulatory oversight ow.ly/9RXL50GEjoT

7:46 PM · Nov 2, 2021

161 See the latest COVID-19 information on Twitter

Read 3 replies

[redacted]

Shame on BMJ for irresponsible reporting.

The serious data problems found with Ventavia's management of Pfizer's vaccine trial:

likely make the vaccine look worse than it actually is

affect 3 of 153 sites

You need to lead with this information, @bmj_latest.
So disappointing.

> **The BMJ** @bmj_latest
> NEW: Our latest investigation hears from a whistleblower engaged in Pfizer’s pivotal covid-19 vaccine trial. Her evidence raises serious questions about data integrity and regulatory oversight ow.ly/9RXL50GEjoT

[redacted]

Well. The problem seems also to be that the whistleblower was fired the day after she alerted the FDA, and the FDA never visited the site. As if there was a Pfizer/FDA collusion. This takes into question the whole trial.

7:21 PM · Nov 3, 2021

147 See the latest COVID-19 information on Twitter

Read 4 replies

[redacted]

A whistleblower involved in Pfizer’s pivotal phase III Covid-19 vaccine trial has “blown the whistle on data integrity issues” in the pharmaceutical giant’s vaccine trial, according to an investigation published in the British Medical Journal (BMJ)

#gript

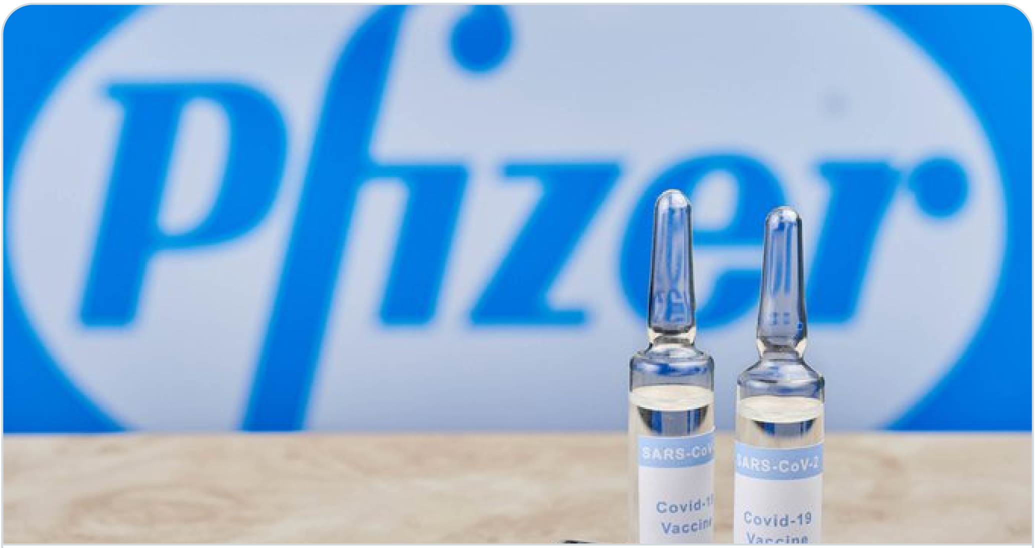

gript.ie
Pfizer Vaccine Trial: 'falsified data and slow to follow up adverse eve...
Data for trial was "crazy mess’

9:42 AM · Nov 3, 2021

146 See the latest COVID-19 information on Twitter

Read 8 replies

[redacted] · Nov 18, 2021

The BMJ published an investigation of Pfizer's vaccine clinical trial bmj.com/content/375/bm...
Ventavia is falsely claiming Brook Jackson never worked on the clinical trial. Her lawyer sent Ventavia a cease & desist for defamation documentcloud.org/documents/2111... /1

BARNES LAW

213.330.3341
601 South Figueroa Street, Suite 4050, Los Angeles, CA 90017
barneslawllp.com

To: Ventavia Research Group & Lauren Foreman
Re: Libel of Brook Jackson
Demand: Retraction
Date: November 11, 2021
Delivery: Personal Service

I write on behalf of Brook Jackson, my client, to demand an immediate retraction and public correction of a false statement made by Ventavia Research Group and Lauren Foreman concerning Brook's work on the clinical trials for the Covid19 vaccine.

On November 5, 2021, Ventavia and Lauren Foreman published a false statement of fact concerning Brook Jackson. As reported in MedPage Today, you stated with regards to Jackson, that "no part of her job responsibilities concerned the clinical trials at issue." This statement is false. This statement impugns the reputation of my client, Brook Jackson, and falsely implies she publicly misrepresented her work on the clinical trials. Point in fact, as you know, Jackson was in fact hired to be the regional director specifically for the "clinical trials at issue," proof of which Jackson provided to MedPage Today following publication of the statement, and which they equally and independently confirmed.

This letter serves as a formal legal demand that Foreman and Ventavia immediately issue a public retraction in a public press release retracting the above statement, further correct the public record by acknowledging "the accuser's job responsibilities did in fact concern the clinical trials at issue", and formally and publicly apologize to my client.

This notice serves as formal notice under Texas statute 73.055, and a failure to timely correct and retract the defamatory statement may result in legal action, including seeking exemplary damages against you for your illicit lies.

[redacted]

Apparently, Ventavia didn't read the story: "Jackson has provided The BMJ with dozens of internal company documents, photos, audio recordings, and emails." bmj.com/content/375/bm... Here's Ventavia's Mercedes Livington invited Jackson a "clean up call" on Pfizer's clinical trial /2

COVID 1001 Clean up Ca

File | Meeting | Help | Acrobat | Tell me what you want to do

Delete | Respond | Meeting Notes | Calendar | Move | Rules | Mark Unread | Categorize | Follow Up | Translate | Find | Related | Select | Read Aloud | Immersive Reader

Delete | Respond | Meeting Notes | Calendar | Move | Tags | Editing | Immersive

Mon 9/21/2020 3:53 PM

ML Mercedes Livingston [redacted]

COVID 1001 Clean up Call

Required William Jones; Kandy Downs; Marnie Fisher; Brook Jackson; Olivia Ray; Kristi Raney

We couldn't find this meeting in the calendar. It may have been moved or deleted.

When Monday, September 21, 2020 4:15 PM-5:00 PM, ~~(Tuesday, September 22, 2020 9:30 AM-10:00 AM)~~ Location https://zoom.us/j/97154812676?pwd=ZEd6QU

Join Zoom Meeting
https://zoom.us/j/97154812676?pwd=ZEd6QUpNdjErbnBDbHI2UUtCY2RDZz09

Meeting ID: 971 5481 2676
Password: 72779673

[illegible]

2:24 PM · Nov 18, 2021

144 See the latest COVID-19 information on Twitter

Read 2 replies

[redacted]

The recent BMJ piece about the Pfizer whistleblower is, in a word, overhyped. There's really nothing there to justify questioning the integrity of the safety data gathered in phase 3, let alone the mountain of data gathered since then.

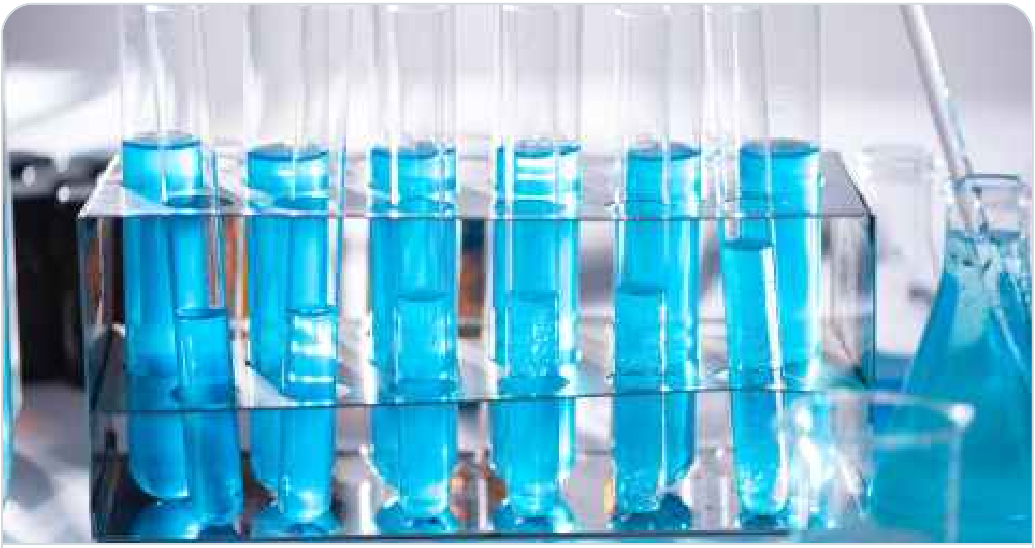

skepticalraptor.com
Pfizer COVID-19 vaccine whistleblower – hand waving about clinical t...
And here we go again – a so-called COVID-19 vaccine whistleblower makes vague claims about the Pfizer clinical trials. Predictably, the ...

6:28 PM · Nov 9, 2021

125 See the latest COVID-19 information on Twitter

Read 3 replies

[redacted]

Have you all seen this yet?
Pfizer research organization falsified data in their Phase III COVID-19 vaccine trial results and fired those who complained about unethical trial practices to the FDA, according to a BMJ investigation, citing a whistleblower.
bmj.com/content/375/bm...

11:44 PM · Nov 2, 2021

Read the full conversation on Twitter

117 See the latest COVID-19 information on Twitter

Read 4 replies

Sputnik V @sputnikvaccine · Nov 2, 2021

BREAKING: Bombshell report in British Medical Journal "raises serious questions" on Pfizer vaccine trial.
BMJ:"Pfizer falsified data, unblinded patients, employed poorly trained vaccinators, was slow to follow up"
Not a word on this in other Western media😳

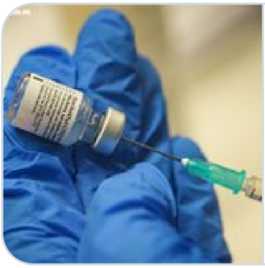

bmj.com
Covid-19: Researcher blows the whistle on...
Revelations of poor practices at a contract research company helping to carry out ...

Sputnik V
@sputnikvaccine

BMJ spoke with Brook Jackson, ex research firm Ventavia executive who informed FDA on Sept 25, 2020 about violations in Pfizer phase 3 COVID vaccine trial.
Other Ventavia employees confirmed her accounts.
Will Western media again ignore stories like this as they did before?

4:39 PM · Nov 2, 2021

117 See the latest COVID-19 information on Twitter

Read 4 replies

[redacted]

FACT:
Associate Editor Peter Doshi, leading medical journal BMJ, writes a scathing criticism of the Pfizer and Moderna vaccine trials.

MAINSTREAM MEDIA:
Crickets.

Same media that trumpeted the "science by press release" marketing.

Journalism is pure propaganda.

thebmjopinion

Access thebmj.com - thebmj

Peter Doshi: Pfizer and Moderna's "95% effective" vaccines—let's be cautious and first see the full data

November 26, 2020

Only full transparency and rigorous scrutiny of the data will allow for informed decision making, argues Peter Doshi

8:02 PM · Dec 13, 2020

[redacted]

Read the full conversation on Twitter

103 See the latest COVID-19 information on Twitter

Read 7 replies

[redacted]

Un'indagine del @bmj_latest ha svelato come il gruppo Ventavia,incaricato da #Pfizer per eseguire i trials di fase III,abbia FALSIFICATO I DATI (e non solo)
Qui la notizia gira da 2gg.. oggi ne parla @Le_Figaro in #Francia.
Qualche giornalONE italiano ne ha fatto cenno??

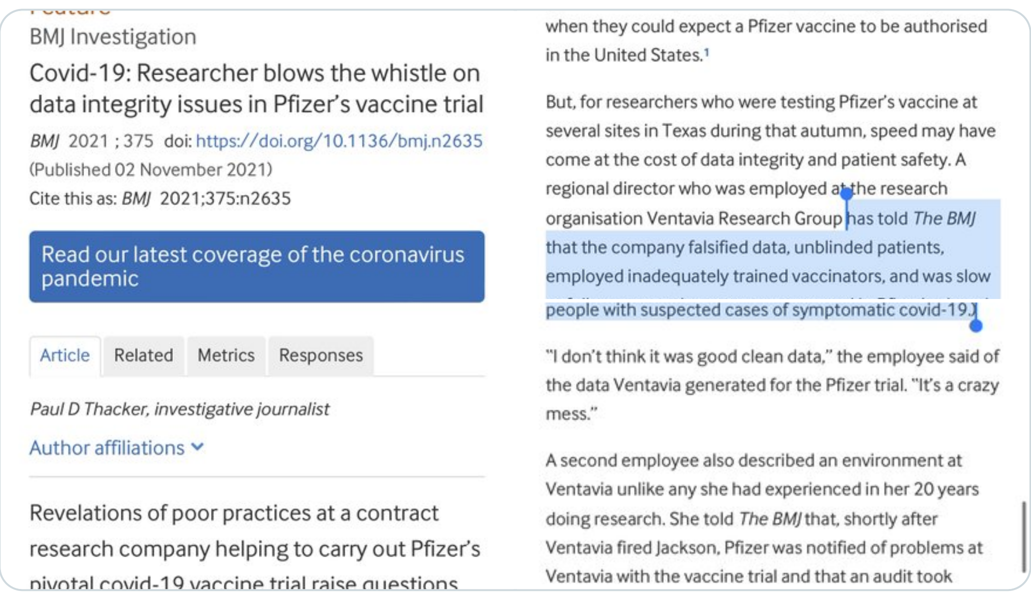

BMJ Investigation

Covid-19: Researcher blows the whistle on data integrity issues in Pfizer's vaccine trial

BMJ 2021 ; 375 doi: https://doi.org/10.1136/bmj.n2635
(Published 02 November 2021)
Cite this as: *BMJ* 2021;375:n2635

Read our latest coverage of the coronavirus pandemic

Article | Related | Metrics | Responses

Paul D Thacker, investigative journalist

Author affiliations

Revelations of poor practices at a contract research company helping to carry out Pfizer's pivotal covid-19 vaccine trial raise questions

when they could expect a Pfizer vaccine to be authorised in the United States.[1]

But, for researchers who were testing Pfizer's vaccine at several sites in Texas during that autumn, speed may have come at the cost of data integrity and patient safety. A regional director who was employed at the research organisation Ventavia Research Group has told *The BMJ* that the company falsified data, unblinded patients, employed inadequately trained vaccinators, and was slow [illegible] people with suspected cases of symptomatic covid-19.

"I don't think it was good clean data," the employee said of the data Ventavia generated for the Pfizer trial. "It's a crazy mess."

A second employee also described an environment at Ventavia unlike any she had experienced in her 20 years doing research. She told *The BMJ* that, shortly after Ventavia fired Jackson, Pfizer was notified of problems at Ventavia with the vaccine trial and that an audit took

Le Figaro @Le_Figaro
Le groupe Ventavia, chargé par Pfizer d'évaluer l'efficacité de son vaccin, a «falsifié des données» et «tardé à assurer le suivi d'effets secondaires», selon un article du British Medical Journal. #COVID19
lefigaro.fr/sciences/covid...

12:46 AM · Nov 4, 2021

100 See the latest COVID-19 information on Twitter

Read 10 replies

[redacted]

‘Where are the FBI this is very disturbing’ #BMJ #Pfizer #Lies #Coverup #Whistleblower

Watch on Twitter

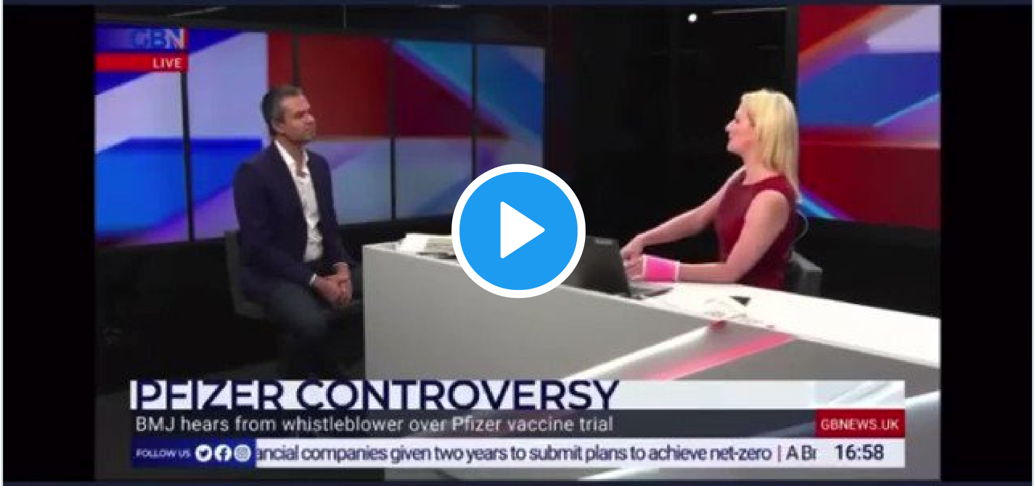

3:59 AM · Nov 6, 2021

95 See the latest COVID-19 information on Twitter

Read 7 replies

[redacted]

🔥BREAKING:🔥

"BMJ whistle blower investigation reveals evidence of falsifying data in pivotal Pfizer Covid vaccine trial "

Dr Aseem Malhotra @DrAseemMalhotra

BREAKING:

BMJ whistle blower investigation reveals evidence of falsifying data in pivotal Pfizer Covid vaccine trial

‘Where is the criminal FBI investigation?’

‘Drug companies paid fines of $13 billion between 2009 - 2014 and nothing has been done to rectify the problem’

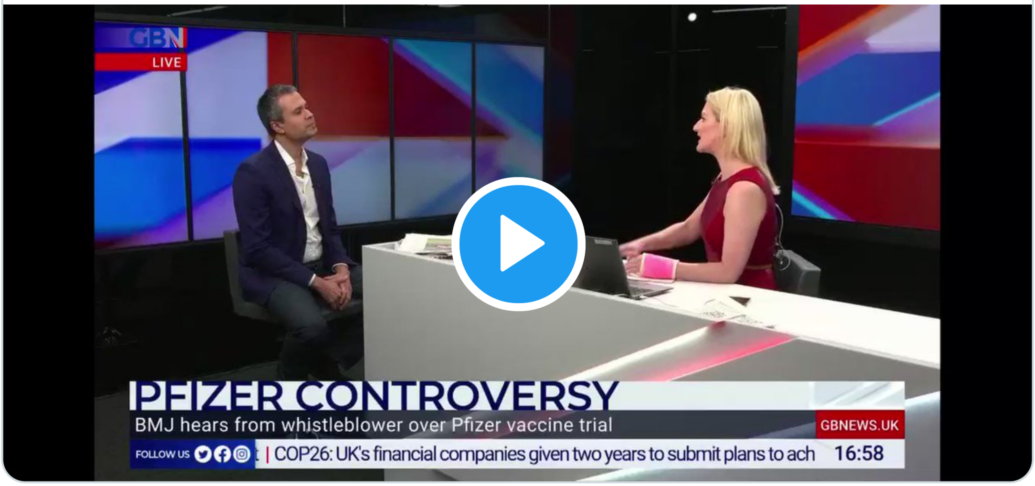

3:29 PM · Nov 5, 2021

92 See the latest COVID-19 information on Twitter

Read 4 replies

[redacted]

Gastei horas vendo no que deu a denúncia de uma ex-funcionária de uma subcontratada (Ventavia) que fez quatro testes locais da vacina da Pfizer. Foi publicada na BMJ, uma agência pré-checa capacha do Zuckerberg chamada Lead Stories rotulou de "sem contexto". A briga continuou...

10:08 PM · Mar 9, 2022

[redacted] Read the full conversation on Twitter

89 See the latest COVID-19 information on Twitter

Read 2 replies

[redacted]

Whistleblower provides evidence of falsified data, unblinded patients and slow follow-up on recording adverse events in Pfizer's pivotal phase 3 trial of Covid mRNA vaccine.
@thackerpd @bmj_latest

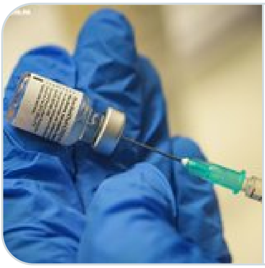

bmj.com
Covid-19: Researcher blows the whistle on data in...
Revelations of poor practices at a contract research company helping to carry out Pfizer's ...

9:44 PM · Nov 2, 2021

83 See the latest COVID-19 information on Twitter

Read 5 replies

[redacted]

This article is about Ventavia, a shitty CRO, and shitty oversight by Pfizer over its CROs, namely this shitty one. It also shows that, since the BMJ is publishing the story, there's no secret cabal stopping major journals from doing anything.

The BMJ @bmj_latest

NEW: Our latest investigation hears from a whistleblower engaged in Pfizer's pivotal covid-19 vaccine trial. Her evidence raises serious questions about data integrity and regulatory oversight ow.ly/9RXL50GEjoT

7:42 PM · Nov 2, 2021

78 See the latest COVID-19 information on Twitter

Read 30 replies

[redacted]

No trust in @pfizer #COVAX data? @bmj_latest calls out flawed #VAX data & supports whistleblower that was fired on day she raised concerns with execs. Same company now running trials on children, pregnant ♀️ & boosters. Why are public not trusting #Pharma?
bmj.com/content/375/bm...

- Participants placed in a hallway after injection and not being monitored by clinical staff
- Lack of timely follow-up of patients who experienced adverse events
- Protocol deviations not being reported
- Vaccines not being stored at proper temperatures
- Mislabelled laboratory specimens, and
- Targeting of Ventavia staff for reporting these types of problems.

Since Jackson reported problems with Ventavia to the FDA in September 2020, Pfizer has hired Ventavia as a research subcontractor on four other vaccine clinical trials (covid-19 vaccine in children and young adults, pregnant women, and a booster dose, as well an RSV vaccine trial; NCT04816643, NCT04754594, NCT04955626, NCT05035212). The advisory committee for the Centers for Disease Control and Prevention is set to discuss the covid-19 paediatric vaccine trial on 2 November.

6:06 AM · Nov 3, 2021

76 See the latest COVID-19 information on Twitter

Read 6 replies

Pfizer UK @Pfizer_UK · Nov 5, 2021

You'll have heard a lot about viruses over the past year, but what exactly are they & how do they make us sick? 🤒

Go behind the science to learn more ⤵️

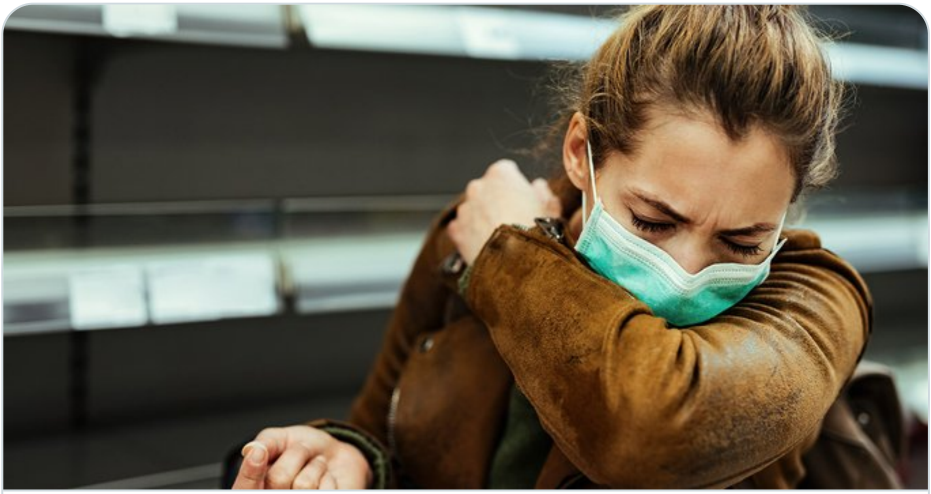

www.pfizer.co.uk

How do viruses make us sick?

[redacted]

Talk us through your rigged vaccine trials, as revealed by the BMJ. I double dare you.

10:53 AM · Nov 9, 2021

♡ 75 See the latest COVID-19 information on Twitter

Read 2 replies

[redacted] Nov 30, 2021

PfizerGate

« ...la FDA a demandé à un juge fédéral américain de faire attendre au public jusqu'en 2076, soit 55 ans, pour divulguer l'ensemble des données et des informations sur lesquelles elle s'est appuyée pour autoriser le vaccin COVID-19 de Pfizer pour adultes. »

5/6

[redacted]

#PfizerGate

« Un autre ancien employé de Ventavia a déclaré au BMJ que Ventavia avait finalement recruté beaucoup plus de participants à l'essai clinique de Pfizer que ceux rapportés à la FDA par Jackson. Ni Pfizer ni Ventavia ne répondront aux questions du BMJ »

6/6

2:13 PM · Nov 30, 2021

73 See the latest COVID-19 information on Twitter

Read 1 reply

[redacted]

Pfizer guilty of fraud, data manipulation and more, according to the British Medical Journal #BMJ whilst reports surge alleging Project Veritas and several journalists have received visits from the #FBI seeking a diary poss c/o Ashley Biden #Pfizergate

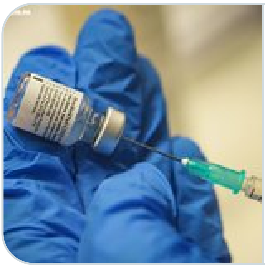

bmj.com
Covid-19: Researcher blows the whistle on data in...
Revelations of poor practices at a contract research company helping to carry out Pfizer's ...

11:59 PM · Nov 5, 2021 from Cheltenham, England

71 See the latest COVID-19 information on Twitter

Read 4 replies

[redacted] · Dec 16, 2021

I find it so strange how some Western leftists seem to want to emphasise "bodily autonomy" over public health and are increasingly buying into weird conspiracy theories about vaccines being some sort of plot.

[redacted]

BMJ Investigation
Covid-19: Researcher blows the whistle on data integrity issues in Pfizer’s vaccine trial

bmj.com/content/375/bm…

Comment: I'm not anti-vax… yet I expect the Cuba Vax was developed more experimentally fastidiously than Pfizer…and others I imagine

✌️ 🇬🇧

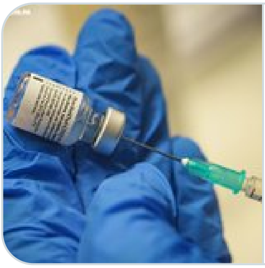

bmj.com
Covid-19: Researcher blows the whistle on data in…
Revelations of poor practices at a contract research company helping to carry out Pfizer’s …

1:15 PM · Dec 16, 2021

67 See the latest COVID-19 information on Twitter

Explore what's happening on Twitter

[redacted]

I'm not surprised but it is so sad that the BMJ article quoting a whistleblower exposing the fact information was falsified in the Pfizer vax trial does not make any MSM. The sleeping cannot be alerted.

8:14 AM · Nov 4, 2021

66 See the latest COVID-19 information on Twitter

Read 3 replies

[redacted]

In light of the alleged Pfizer fraud reported in the BMJ, sixteen doctors and researchers in Sweden have called for Covid-19 vaccination to be paused pending risk/benefit analyses for all age groups. @indepdubnrth @dr_morrissey @BillyRalph @DrAnneDerry @reasonoverfear

> [redacted]
>
> "Press release: Research fraud at Pfizer subcontractor. Now 16 doctors and researchers are calling for vaccination in Sweden to be paused."
> lakaruppropet.se/wp-content/upl...
> "The British Medical Journal (BMJ) reveals on 2 November 2021 that a subcontractor to Pfizer is likely to have...

2:11 PM · Nov 7, 2021

68 See the latest COVID-19 information on Twitter

Explore what's happening on Twitter

[redacted]

Newsweek hit on Fauci and the BMJ Pfizer 'revelations' on the same day: tactical retreats compelled by the sustained mass mobilization, protests big and small, walkouts, constant 'emergency rule' breaking, massive public exposure of scientific fraud, jab injuries & deaths.

5:39 PM · Nov 2, 2021

67 See the latest COVID-19 information on Twitter

Read 4 replies

[redacted]

A researcher with @pfizer contractor @VRG_FortWorth blows the whistle about #COVID19 mRNA vaccine clinical trials' standards. Not the kind of news you want to read about a jab being now given to many millions people worldwide. And yet. @thackerpd for @bmj_latest #whistleblowing

[redacted]

Covid-19: Researcher blows the whistle on data integrity issues in Pfizer's vaccine trial bmj.com/content/375/bm...
Revelations of poor practices at research company helping carry out Pfizer's pivotal #COVID19 vaccine trial raise questions of data integrity & regulatory oversight

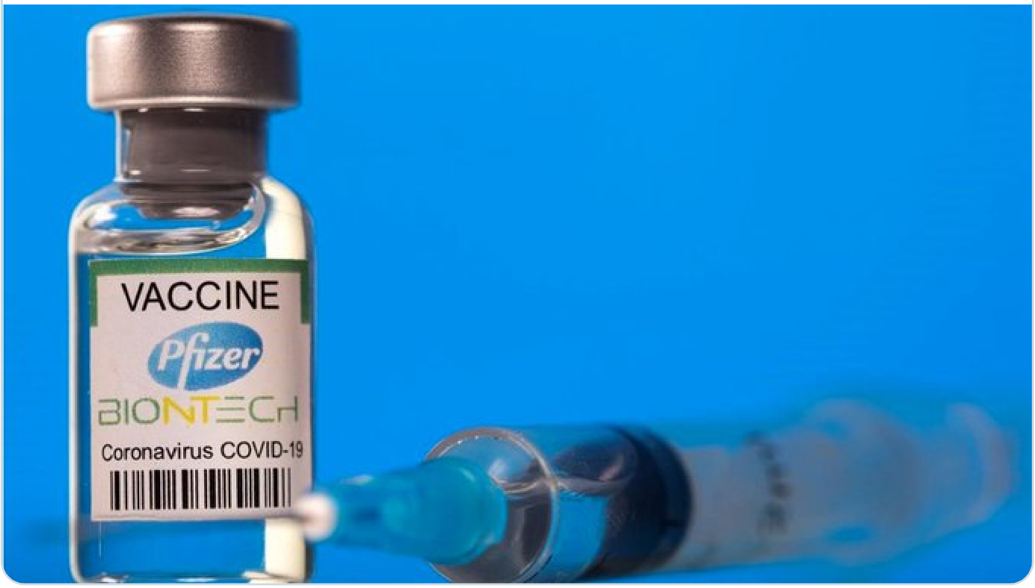

2:54 PM · Nov 2, 2021

65 See the latest COVID-19 information on Twitter

Read 5 replies

[redacted]

In late 2021, whistle-blower Brook Jackson went public with allegations of ‘falsified data’ in Pfizer's mRNA trial, to the BMJ.

Meet Brook & Molecular Biologist Dr Jessica Rose tomorrow night on Making Sense...with Sara Haboubi, 7pm GMT 12th April.

bit.ly/makingsense05

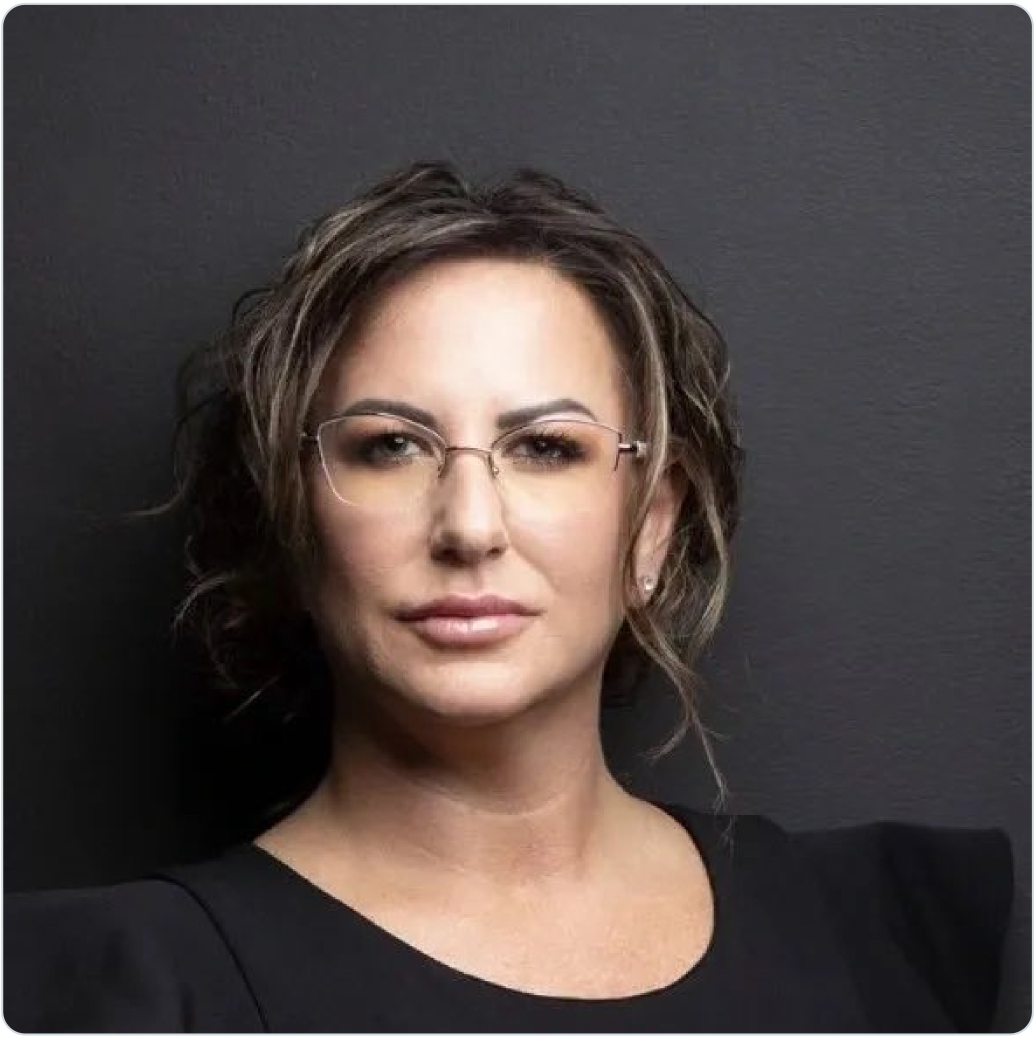

9:00 PM · Apr 11, 2022

57 See the latest COVID-19 information on Twitter

Read 3 replies

[redacted] · Nov 29, 2021

Recently, several authors with track records of vaccine hostility have had features in @bmj_latest. I took a look at the alt-metrics of all features in 2021; articles critical of vaccination garner HUGE traction, giving a dangerous veneer of legitimacy to nonsense. Thread 1/n 🧵

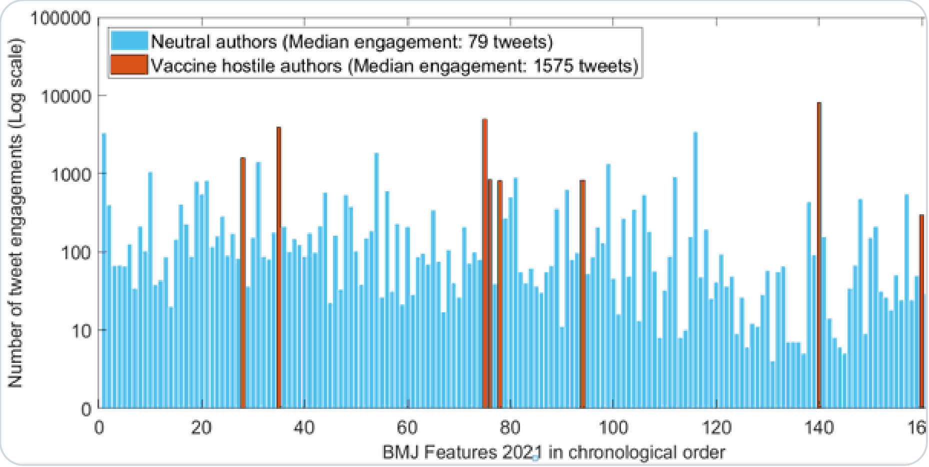

[redacted]

Some background: recently BMJ published a piece by Thacker, which alleged data fraud by Pfizer. It was unmitigated nonsense, and textbook misrepresentation. Still, the piece went viral with over 86k tweets. @gorskon writes on it here for reference (2/n)

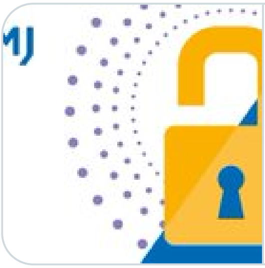

sciencebasedmedicine.org
What the heck happened to The BMJ?
Last week, The BMJ published an "exposé" by Paul Thacker alleging patient unblinding, data ...

12:03 PM · Nov 29, 2021

55 See the latest COVID-19 information on Twitter

Read 8 replies

[redacted]

Given how many people worldwide are expected to be vaccinated, you'd expect gold standards to be applied in trials

ThIs from BMJ suggests otherwise...

'Researcher blows the whistle on data integrity issues in Pfizer’s vaccine trial'

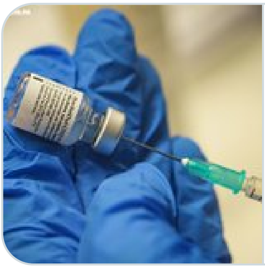

bmj.com
Covid-19: Researcher blows the whistle on data in...
Revelations of poor practices at a contract research company helping to carry out Pfizer’s ...

3:52 PM · Nov 2, 2021

55 See the latest COVID-19 information on Twitter

Read 4 replies

[redacted]

Facebook is censoring the British Medical Journal. They wrote on the flawed trials of Pfizer vaccines & anyone posting it on Facebook failed or had a warning attached.

The "fact check" was seen by the BMJ to be "inaccurate, incompetent & irresponsible.”

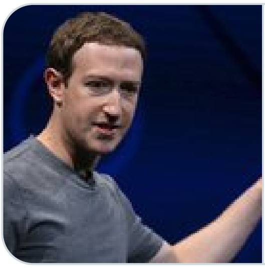

newyorknationalreview.com
Facebook Now Censoring the British Medical Jour...
Facebook Now Censoring the British Medical Journal Because New Science Goes Against Pfizer’...

11:30 AM · Dec 26, 2021

50 See the latest COVID-19 information on Twitter

Read 1 reply

[redacted] Nov 2, 2021

Replying to @bmj_latest

Ok, someone alleges that a site (which handled 4% of the subjects) *may* have had some paperwork left out that could *possibly* unblind the investigators for some subjects. And you're running with THAT headline?

[redacted]

1/2 The problem with @pfizer is that they are repeat offenders all over the world 🇺🇸 🇬🇧 🇳🇬 🇨🇳 and others for forgery, falsification of experimental results, unjustified exaggeration of the pricing of its products, endangering lives on uncontrolled trials on children in 🇳🇬

7:34 PM · Nov 2, 2021

50 Reply Copy link

Read 1 reply

[redacted]

Pfizer vaccine trials whistleblower interviewed by the bmj.

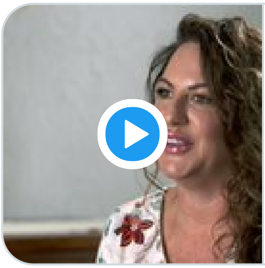

youtube.com

Covid-19: Researcher blows the whistle on data in...

For researchers who were testing Pfizer's vaccine at several sites in Texas during that autumn, spee...

11:54 PM · Mar 23, 2022

50 See the latest COVID-19 information on Twitter

Read 1 reply

[redacted]

Essential viewing: @YoureAllDunces speaks with Pfizer whistleblower Brook Jackson, who recently exposed Pfizer/Ventavia's rampant clinical-trial malfeasance to @bmj_latest.

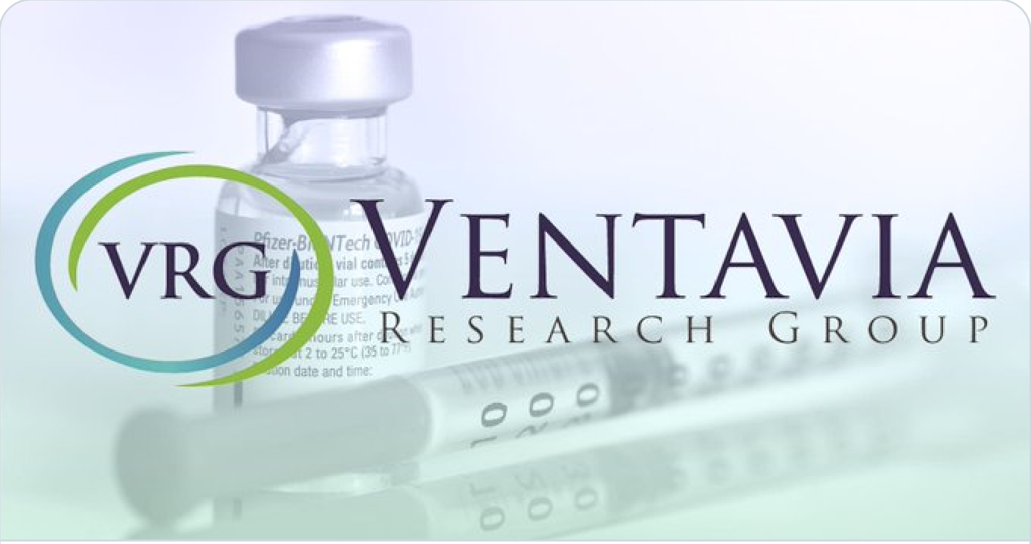

thelastamericanvagabond.com
Brook Jackson Interview - Pfizer Whistleblower Exposes Cover Up C...
Joining me today is Brook Jackson, the Pfizer whistleblower who recently spoke with the British Medical Journal revealing documente...

12:15 AM · Dec 3, 2021

46   See the latest COVID-19 information on Twitter

Explore what's happening on Twitter

Source Classifications

Citing sources were manually classified according to strict definitional criteria. The resultant classifications and sorting criteria are

News media sources

Registered Media outlets

20 minutes, 6 Minutes, 7News, ABC.net.au, Aftenbladet, Augsburger Allgemeine, BFMTV, Big News Network, Brasil 247, CNEWS Matin, *CORRECTIV,* Dagbladet, Dagens.dk, Daily Mail, Deutsches Ärzteblatt, Die Rheinpfalz, Die Welt, El Comercio, Express Informer, Fanpage, Foreign Affairs New Zealand, FranceTV Info, Gazeta Do Povo, Giornalettismo, Heise, Herald Scotland, Il Foglio, Impronta Unika, Kleine Zeitung, L'Express, L'independent, La Dépêche, La Voix du Nord, Le Figaro, Le Monde, Le Parisien, Libération, MedPage Today, Medical Republic, MedicalXpress, Medium US, Medscape, Meduza, Midi Libre, Mitteldeutscher Rundfunk, Moustique, N-TV, NZCity, Neue Zürcher Zeitung (NZZ), New Statesman, New Zealand Herald, News.am, Newsbreak, Pagina 12, Pediatric News, Pharmazeutische Zeitung, Potsdamer Neueste Nachrichten, Pourquoi Docteur, Quotidiano Net, RTBF, Redacción Médica, Rhein-Neckar-Zeitung, SPIEGEL ONLINE , Sciences et Avenir, Scoop, Stern, Tagesschau, The Cairns Post, The Conversation, The Print, The Wire, Timeturk, Top Santé, Zougla, der Freitag, Marianne

News Aggregators

Websites relaying content from other sources without additional independent reporting (counted by Altmetric as a media source)

BizNews.com

Yahoo! News Brazil

Yahoo! News

MSN

Big News Network

Tek Deeps

TechFeedThai

Express Informer

DNYUZ

Pressfrom

Foreign Affairs New Zealand

ZAP

Free

Non-media media

Conspiracy sites

Sites were checked manually to see their editorial portfolio, and also assessed for quality by NewsGuard / Media Bias-factcheck. The sites deemed conspiracy sites are listed below, along with the factors that led to this designation.

Globalresearch.ca - Long established conspiracy website established in 2001 by Michel Chossudovsky. Accused of information warfare by NATO's Strategic Communications Centre of Excellence (STRATCOM), identified by US State department as being a proxy for a Russian disinformation campaign (the site was described as ""key accelerant role in helping popularize articles with little basis in fact that also happen to fit the narratives being pushed by the Kremlin"). Derided by PolitiFact as having *"..advanced specious conspiracy theories on topics like 9/11, vaccines and global warming"*.

Epoch Times – A far-right media organ associated with Falun Gong religious movement, which promotes far-right politicians across Europe, and was the second-largest funder of pro-Trump Facebook advertising after the Trump campaign in 2016. The Epoch Media Group's sites spread conspiracy theories promoting ideas such as QAnon and anti-vaccine misinformation, and false claims over the 2020 US election. Described by the New York Times as a "global-scale misinformation machine"

Zero hedge – Originally a libertarian investment site, Zero Hedge expanded into non-financial political content, including conspiracy theories on vaccination and the origins of COVID-19, and radical right, alt-right, and pro-Russia positions. This non-financial commentary led to multiple site bans by global social media platforms, although bans on Facebook and Twitter ban were later reversed. In house content is posted under the pseudonym "Tyler Durden", with the founder and main editor identified as Daniel Ivandjiiski.

France-Soir – Once a physical format newspaper in the 1950/1960s, this French tabloid re-emerged in online only format in 2016. Since then ,it has promoted conspiracy theories and was derided by NewsGuard for "failing to adhere to several basic journalistic standards". The site has repeatedly been criticized for amplifying baseless conspiracy theories.

Breitbart News – An alt-right centred site since 2007, it has repeatedly aired conspiracy theories and intentionally misleading stories. The site has repeatedly platformed COVID-19 disinformation, including falsehood about a cure, leading to restrictions from major social media outlets for sharing debunked and dangerous fictions.

Sign of the Times (SOTT.NET) – Founded in 2002 in North Carolina by a fringe religious movement, the site is rated by mediabias / factcheck as "Right Biased and a strong Conspiracy and Pseudoscience website based on the promotion of false, unproven, or misleading information that cannot be verified or has been debunked." It is also rated negatively by NewsGuard.

Trialsite news - Anti-vaccine centred site with history of pushing vaccine negative information. The site repeatedly posts false information on COVID-19, leading to several failed factchecks by media bias / factcheck and the conclusion " Overall, we rate TrialSite News a strong Pseudoscience source based on promoting misleading and false claims regarding Covid-19 vaccines."

WorldNetDaily (WND) - American far-right health disinformation vector known for promoting falsehoods and conspiracy theories. Described by Southern Poverty Law Center of being a major source of "anti-government conspiracy theories, gay-bashing, anti-Muslim propaganda, and End Times prophecy"

The Blaze / BlazeMedia – American conservative media conspiracy formed in 2018, originally an outfit for Glenn Beck. Noted for disseminating conspiracy theories and failing factchecks, rated by media bias / fact checker as "strongly Right Biased and Questionable based on the promotion of conspiracy theories and numerous failed fact checks."

Fria Tider (Free Times) –Extreme in views and operating out of Estonia, the site pushes racist anti-immigration conspiracy theories. The New York Times list it as among one of four prominent far-right sites, writing that, "Fria Tider is considered not only one of the most extreme sites but also among the most Kremlin-friendly. It frequently swaps material with the Russian propaganda outlet Sputnik. The site is linked, via domain ownership records, to Granskning Sverige, called the Swedish "troll factory" for its efforts to entrap and embarrass mainstream journalists. Among its frequent targets: journalists who write negatively about Russia." Rated by fact-checker as "extreme right biased and Questionable based on the use of poor sources who routinely fail fact checks, promotion of conspiracy theories and propaganda".

Other citing non-news sites deemed conspiracy pages for lacking any clear editorial information required for media outlets, and pushing other debunked conspiracy theories on human inspection

Jackpine radicals

Achgut.com

Civil.de

Russian state agencies / aligned

Sources with a .ru web address were deemed Russian state aligned media services, as all media in Russia is Kremlin controlled. RT Network have non .ru websites, but are well-known vectors of Russian state disinformation and a well-established propaganda outfit.

RT Network

Lenta.ru

Russian News Agency (TASS)

RBC Daily (RBC.RU)

MedPortal.ru

Gazeta.ru

Popmech.ru (Also known as Techninsider.ru)

Extended material

Extended Rationale for classification of article as malinformation

The article in question, entitled "Covid-19: Researcher blows the whistle on data integrity issues in Pfizer's vaccine trial", was published as an investigative piece, based on an account from a whistleblower briefly employed in a managerial capacity at Ventavia Research Group (VRG), a Texas-based contract research organization (CRO) hired by Pfizer to help run its phase 3 randomized controll trial (RCT) of its then-investigational mRNA-based COVID-19 vaccine BNT162b2. VRG ran three sites in Texas (out of 153 total sites worldwide) recruiting subjects in 2020. The reporting accused VRG of severe problems and violations of FDA regulations, including serious charges including unblinding of subjects and data falsification.

Serious issues with this reporting were detected almost immediately after publication by independent fact-checks (Miller 2022) , medical press (Clark 2022) and by medical bloggers (Gorski 2021),. These criticisms focused chiefly on the paucity of evidence presented to support such bold conclusions, which were largely speculative and extrapolated unverified reported problems at VRG to the entire multicenter clinical trial, callings its results into question. The most incendiary charge, data falsification, was reported with no evidence to support it beyond an ambiguous email (dated before the whistleblower's hiring) including the text "Go over e-diary issue/falsifying data etc,", and noting that an employee had been "verbally counselled for changing data and not noting late entry". Neither incident, however, demonstrates intent to falsify data, and a less uncharitable reading would be that employees were correctly instructed that clear audit trails are indispensable to trial integrity. Despite a clear claim that VRG had "falsified data", the article provided no evidence that any data falsification had ever occurred at VRG or elsewhere.

The second most serious charge was that clinical trial participants had been unblinded at the three Texas sites managed by VRG, where it was speculated that the potential for participants to become unblinded existed. This charge was based on a photograph provided showing "vaccine packaging materials with trial participants' identification numbers written on them left out in the open, potentially unblinding participants" and an observation that drug assignment confirmation printouts had been "left in participants' charts, accessible to blinded personnel," the latter problem corrected two months into the trial. The article nonetheless extrapolated from the possibility that inadvertent unblinding might have occurred in this instance to speculate that "unblinding may have occurred on a far wider scale", despite presenting no evidence that any actual unblinding had occurred at any of the three VRG sites in Texas, much less at any other Pfizer trial site worldwide.

There were other misleading aspects to the report as well; for example, an assertion that full swabs were not taken from 477 people with suspected COVID. This figure however refers to the entire clinical trial involving 42,436 people in 153 sites around the world, not just the three VRG centers in Texas. Worldwide, 4,931 participants developed protocol-defined symptoms within 7 days of their second dose, PCR tests were performed for all but 477 (9.7%) of this pool, comprising 210 in the trial arm (4.3%) and 267 in the control arm (5.4%). The percentage of non-tested trial participants in the active arm (9.2%) to the control arm (10.1%) was similar. FDA clinical reviewers explicitly examined this issue in a sensitivity analysis and concluded that "missing PCR results would not have a significant effect on the vaccine efficacy results" (FDA 2021). This critical context was not provided in the article.

Overall, the narrative, presented as factual reporting from an investigative journalist, implied that the problems allegedly uncovered at VRG were generalizable to the entire RCT. The report also

included a discussion of the "history of lax oversight" by the FDA of clinical trials undertaken to win licensure of experimental therapeutics. Although the problems attributed to it in the report were undeniably possible failings of VRG (albeit not demonstrated by the evidence presented in the article), the article generalized the problems claimed to have been found at VRG to the entire Pfizer trial 24 times. In isolation, errors or misunderstandings alone might render such an article as misinformation, but in this instance these errors went beyond misunderstanding, instead making assertions likely to be harmful to public perception of Pfizer and of vaccine science more broadly.

Accordingly, the article as it stands fits the European Council definition of malinformation ("information that is based in reality, but is used to inflict harm on a person, organisation or country"), precisely because it uses real potential issues with a single CRO contracted by Pfizer as a basis for unevidenced claims that Pfizer "falsified data" and "unblinded patients", despite such assertions being, at best speculative extrapolation and, at worst, completely unfounded. As this article has by far the highest Altmetric score (45,800 at time of analysis) of any article in a biomedical journal, (or any other scientific work in any field) ever published, analyzing its propagation and reception is critical to ascertain impact on public perception.

Extended Discussion

In this case, this poor understanding manifested itself in how journal editors promoted the article and then reacted to criticisms of its methods and findings. Specifically, the issue in which the story appeared promoted the article with an accompanying editorial that went beyond the initial article, extrapolating problems alleged at VRG not just to the entire Pfizer clinical trial but to pharmaceutical company clinical trials in general (Godlee 2021). After the story had gone viral, it was independently fact-checked by Lead Stories at the behest of Meta (parent company of Facebook), resulting in shares of the article on Facebook being tagged as: "Missing context ... Independent fact-checkers say this information could mislead people." Lead Stories' (Miller 2022) labelling was prompted by recognition that Mr. Thacker's article was potentially malinformation, and observations of how the article was being misinterpreted, drawing conclusions similar to the conclusions of our analysis presented in this work.

In response to fact-checking, rather than addressing the specific criticisms of the article's find-ings, the journals' editors published an open letter (Godlee and Abbasi 2021) to Meta CEO Mark Zuck-erberg charging that Lead Stories had failed to "identify anything false or untrue" while citing the journal's "legal review, external peer review and…usual high level editorial oversight and review" of the manuscript, thus demonstrating their lack of understanding of malinformation, which by definition consists of verifiable, ostensibly factual information presented in a manner that can mislead. The same open letter also criticized the fact checkers, arguing that, rather "than investing a proportion of Meta's substantial profits to help ensure the accuracy of medical information shared through social media, you have apparently delegated responsibility to people incompetent in carrying out this crucial task." Elsewhere, an associate editor defended the journal (McCreary 2022), stating that the story was "subject to the same rigorous peer review that scientific studies published." Unfortunately, this reaction only reinforces our argument that inoculation and prebunking of malinformation are important not just for the public, but for journal editors and reviewers as well.

These responses to fact-checking missed the crux of the problem of malinformation, specifically that malinformation misleads not through the falsity of any individual finding reported, but rather through how findings are woven together with various "facts," often poorly supported by evidence but not demonstrably false, together into a misleading tapestry. For that reason, in our case study,

“rigorous peer review” appears to have been ill-equipped to detect malinformation. We conclude that such the publication of this story and the reactions of the editors to criticism based on fact-checking likely reflect an all-too common lack of understanding of the difference between misinformation and malinformation. We cannot speak to the intentions of either the journal nor author, especially as malinformation can arise both intrinsically and extrinsically. In the latter scenario, cherry-picked factlets can render authors and journals victims of malinformation as in addition to vectors.

References

Clark C. Experts Blow Whistle on Alleged COVID Vaccine Whistleblower Claims. *MedPage Today,* 2022. Retrieved 28 May 2022, from Medpage

Food and Drug Administration (FDA), Clinical Review Memo, August 2021. Available online

Godlee F. Covid 19: A strong pandemic response relies on good data. *BMJ* 2021, 375:n2668.

Godlee F, Abbasi K. Rapid Response: Open letter from The BMJ to Mark Zuckerberg. *BMJ*. 2021;375:n2635.

Gorski, D “What the heck happened to the BMJ?” - Science Based medicine, November 2021, Available online at https://sciencebasedmedicine.org/what-the-heck-happened-to-the-bmj/

McCreary, J. Fact check: Report questioning Pfizer trial shouldn’t undermine confidence in vaccines. *CBS News*, 2021. Retrieved 28 May 2022, from CBS

Miller D. Fact Check: The British Medical Journal Did NOT Reveal Disqualifying And Ignored Reports Of Flaws In Pfizer COVID-19 Vaccine Trials. *Lead Stories*, 2022. Retrieved 28 May 2022, from Lead stories
